# Regulation of transcription by the Arabidopsis UVR8 photoreceptor involves a specific histone modification

**DOI:** 10.1007/s11103-016-0522-3

**Published:** 2016-08-17

**Authors:** Christos N. Velanis, Pawel Herzyk, Gareth I. Jenkins

**Affiliations:** 1 Institute of Molecular, Cell and Systems Biology, College of Medical, Veterinary and Life Sciences, University of Glasgow, Bower Building, Glasgow, G12 8QQ UK; 2 Glasgow Polyomics, Wolfson Wohl Cancer Research Centre, University of Glasgow, Garscube Estate, Switchback Road, Bearsden, G61 1QH UK

**Keywords:** UVR8, UV-B, Histone modification, HAT inhibitors, Transcription, Chromatin, *Arabidopsis thaliana*

## Abstract

The photoreceptor UV RESISTANCE LOCUS 8 (UVR8) specifically mediates photomorphogenic responses to UV-B wavelengths. UVR8 acts by regulating transcription of a set of genes, but the underlying mechanisms are unknown. Previous research indicated that UVR8 can associate with chromatin, but the specificity and functional significance of this interaction are not clear. Here we show, by chromatin immunoprecipitation, that UV-B exposure of Arabidopsis increases acetylation of lysines K9 and/or K14 of histone H3 at UVR8-regulated gene loci in a UVR8-dependent manner. The transcription factors HY5 and/or HYH, which mediate UVR8-regulated transcription, are also required for this chromatin modification, at least for the *ELIP1* gene. Furthermore, sequencing of the immunoprecipitated DNA revealed that all UV-B-induced enrichments in H3K9,14diacetylation across the genome are UVR8-dependent, and approximately 40 % of the enriched loci contain known UVR8-regulated genes. In addition, inhibition of histone acetylation by anacardic acid reduces the UV-B induced, UVR8 mediated expression of *ELIP1* and *CHS*. No evidence was obtained in yeast 2-hybrid assays for a direct interaction between either UVR8 or HY5 and several proteins involved in light-regulated histone modification, nor for the involvement of these proteins in UVR8-mediated responses in plants, although functional redundancy between proteins could influence the results. In summary, this study shows that UVR8 regulates a specific chromatin modification associated with transcriptional regulation of a set of UVR8-target genes.

**Electronic supplementary material:**

The online version of this article (doi:10.1007/s11103-016-0522-3) contains supplementary material, which is available to authorized users.

## Introduction

Plants are immobile autotrophs whose optimal growth and development relies heavily on light. In addition to its role as an energy source, light provides regulatory signals for development throughout the plant life cycle (Kami et al. 2010). Ultraviolet-B light (UV-B; 280–315 nm) is a minor component of the solar spectrum, but regulates several aspects of plant development (Jenkins 2009; Jansen and Bornman 2012). The only known UV-B photoreceptor is UV RESISTANCE LOCUS 8 (UVR8), which employs a unique photosensory mechanism for light absorption and initiation of the signalling events that lead eventually to particular physiological responses (Jenkins 2014a, b; Ulm and Jenkins 2015).

Structural characterization of UVR8 (Christie et al. 2012; Wu et al. 2012) revealed that it does not employ an external chromophore, but instead uses intrinsic tryptophan amino acids for UV-B photoreception (Rizzini et al. 2011; O’Hara and Jenkins 2012; Mathes et al. 2015; Wu et al. 2015). Key steps in photomorphogenic UVR8-dependent signalling have been elucidated and can be summarised as follows: UV-B causes monomerisation of the UVR8 homodimer (Rizzini et al. 2011), rapid accumulation of the photoactivated monomers in the nucleus (Kaiserli and Jenkins 2007), and reorganisation of nuclear COP1-SPA-containing complexes favouring COP1-SPA association with UVR8 monomers (Favory et al. 2009; Cloix et al. 2012; Huang et al. 2013, 2014; Yin et al. 2015). UVR8-COP1-SPA complexes positively regulate the transcription of target genes, among which are *HY5* and *HYH*, whose products in turn control the expression of many downstream genes that mediate UVR8-dependent responses (Brown et al. 2005; Oravecz et al. 2006; Brown and Jenkins 2008; Favory et al. 2009). A negative regulatory feedback loop is established (Gruber et al. 2010), as RUP1 and RUP2 accumulate following UVR8- and HY5-mediated transcriptional stimulation of their expression and repress the pathway by competing with COP1-SPA for binding to the C-terminal region of UVR8 and by facilitating the regeneration of the UVR8 homodimer (Heijde and Ulm 2013; Heilmann and Jenkins 2013).

However, the events that follow the interaction of UVR8 with COP1-SPA and lead to the transcriptional activation of target genes remain obscure. It is well established that HY5 and HYH, acting in partial redundancy, are the two major transcriptional effectors downstream of UVR8 (Brown et al. 2005; Oravecz et al. 2006; Brown and Jenkins 2008; Favory et al. 2009; Stracke et al. 2010; Feher et al. 2011; Huang et al. 2012). HY5 is post-translationally stabilised by UV-B (Favory et al. 2009; Huang et al. 2013) and acts as a central player of a positive feedback loop by promoting the expression of both its own gene (Abbas et al. 2014; Binkert et al. 2014) and *COP1* (Huang et al. 2012). There is evidence that UVR8 may act at the level of chromatin. UVR8 is detected in isolated chromatin and binds to histones in vitro, preferentially to H2B (Brown et al. 2005; Cloix and Jenkins 2008). Furthermore, the in vivo detection of UVR8 on plant chromatin has been reported using chromatin immunoprecipitation (ChIP) assays (Brown et al. 2005; Kaiserli and Jenkins 2007; Cloix and Jenkins 2008; Favory et al. 2009; Cloix et al. 2012). Intriguingly, the binding of UVR8 to chromatin is observable regardless of UV-B illumination, but it has been argued that without quantitative data it is not possible to determine whether UV-B stimulates this phenomenon (Jenkins 2014a). Although it is conceivable that UVR8 might appear on chromatin as a member of a multipartite protein complex, currently no experimental data are available in support of such a view. A candidate that has been investigated in that respect is COP1, but it appears to be dispensable for UVR8-chromatin association (Favory et al. 2009; Cloix et al. 2012). Moreover, chromatin binding appears only for a subset of the genetic loci occupied by UVR8-regulated genes, and although the association has been detected on promoter regions, it appears not to be restricted to them. For *HY5* particularly, the UVR8-chromatin interaction spans the entire locus, covering promoter, coding and 3′ non-coding regions (Cloix and Jenkins 2008). These data have been interpreted to imply that UVR8 might be directly involved in promoting gene expression by participating in processes that keep chromatin in a transcriptionally active euchromatic conformation. Indeed, it is conceivable that UVR8, by associating with chromatin on target loci, could act as a recruiting agent for protein complexes with histone modifying or chromatin remodelling activity. Conversely, it is important to keep in mind that the association of UVR8 with chromatin might be non-specific, resulting from UVR8’s ability to stick to histones during chromatin isolation. Binkert et al. (2016) recently questioned the *in vivo* UVR8-chromatin association after failing to detect UVR8 on certain loci, including *HY5*.

For more than two decades, it has been known that chromatin-level mechanisms are of utmost importance for the proper manifestation of various light responses (Fisher and Franklin 2011; Barneche et al. 2014; Wu 2014). Several studies have documented light-mediated alternations of chromatin between its two main compaction states, euchromatin and heterochromatin (Tessadori et al. 2007, 2009; van Zanten et al. 2010; Bourbousse et al. 2015), and diverse types of post-translational histone modifications have been reported to contribute in facilitating the appropriate transcriptional outputs (Chua et al. 2001, 2003; Offermann et al. 2006, 2008; Guo et al. 2008; Charron et al. 2009; Jang et al. 2011; Jing et al. 2013; Bourbousse et al. 2015). The Cry2 photoreceptor has been suggested to be involved in heterochromatin decondensation under low light conditions (van Zanten et al. 2010) and GFP-CRY2 fusion protein has been detected on anaphase chromosomes (Cutler et al. 2000). This has been interpreted to indicate that cry2 could influence histone-based processes directly or through interactions with transcription factors such as HY5 or CRYPTOCHROME-INTERACTING BASIC-HELIX-LOOP-HELIX (CIBs) (van Zanten et al. 2010). Studies in maize, and Arabidopsis, have highlighted the significance of UV-B-mediated chromatin-based processes coupled to transcriptional regulation (Casati et al. 2006, 2008; Cloix and Jenkins 2008; Campi et al. 2012). Hence it would be valuable to pinpoint those modifications that are physiologically significant for photomorphogenic UV-B responses, and to assess how, if at all, their appearance is regulated by UVR8. Here we show that a particular chromatin modification is associated with the regulation of transcription mediated by UVR8.

## Materials and methods

### Plant material and treatments

The wild-type (WT) *Arabidopsis thaliana* ecotypes used in this study were Landsberg *erecta* (L*er*), Columbia (Col-0) and Wassilewskija (W*s*), seeds of which were obtained from The European Arabidopsis Stock Centre (NASC, Nottingham, UK). Prof. Daniel Kliebenstein (UC Davis, CA, USA) provided the *uvr8-1* (L*er*) mutant seeds. The *hy5-ks50*/*hyh* and *hd1* mutants (W*s*) were supplied by Prof. Xing-Wang Deng (Yale University, CT, USA) and Professor Jeffrey Chen (University of Texas, Austin, USA) respectively. Seeds of the T-DNA insertional mutants were obtained from NASC, with the following accession numbers and parent lines: *gcn5*, N674989 SALK_048427; *hac5*, N667192 SALK_122443; *taf1*, N660015 SALK_088103; *fve*, N878321SAIL_1167_E05. The *gcn5, fve* and *hd1* alleles have been described previously (Long et al. 2006; Pazhouhandeh et al. 2011; Tian et al. 2003 respectively).

Plants were grown in compost in small pots, with multiple individuals in each pot. Thus, each harvested sample included material from at least 10 plants (for each gene expression treatment) or at least 200 plants (for each ChIP sample).

For gene expression studies, plants grown for 3 weeks under constant white light (warm white fluorescent tubes, Osram; 60 μmol m^−2^ s^−1^) were placed in darkness overnight and then illuminated either with 1.5 μmol m^−2^ s^−1^ narrowband UV-B light (Philips TL20W/01RS; spectrum shown in Cloix et al. 2012) for 3 h or low fluence rate white light (LW; warm white fluorescent tubes, Osram; 15 μmol m^−2^ s^−1^) for 3 h (as controls). After treatment, leaf tissue was collected, snap frozen in liquid nitrogen and stored at −80 °C.

For ChIP experiments, plants were grown under low fluence rate white light (15 μmol m^−2^ s^−1^) from germination and the light treatments were identical to the aforementioned, except that no overnight dark treatment was applied, the duration of illumination was 4 h and, upon completion of treatment, leaf tissue was collected and kept on ice until fixation.

For hypocotyl elongation assays, sterilized, cold-stratified seeds were germinated under low fluence rate white light (1.5 μmol m^−2^ s^−1^) without any measurable UV-B (control plants), or with supplementary 1.5 μmol m^−2^ s^−1^ narrowband UV-B. Hypocotyl lengths from at least 25 seedlings were measured 5 days after germination and results were presented as mean values ± SE. From the remaining seedlings of each treatment, protein was extracted and immunoblots detecting expression levels of CHS were performed.

### Protein gel-blot, RT-PCR and yeast 2-hybrid assays

Protein extraction from Arabidopsis seedlings and immunoblots were performed as described in Cloix and Jenkins (2008) using antibodies presented in Supplementary Table S1. RT-PCR analysis was performed as in Brown and Jenkins (2008) with primers presented in Supplementary Table S2. Yeast-two hybrid assays were performed exactly as described in Hayes et al. (2014) unless otherwise stated.

### ChIP analyses

ChIP assays were performed as described in Cloix and Jenkins (2008) with antibodies presented in Supplementary Table S1. The immunoprecipitated DNA was analysed via quantitative real-time PCR (Applied Biosystems StepOnePlus) employing absolute standard curve-mediated quantification. To that end, the PCR products of each primer pair (Supplementary Table S2), targeting either a promoter region encompassing the TSS or a downstream transcribed region of the genetic loci of interest, were subcloned in the pCR2.1 TOPO vector (Life technologies K4550-40) according to the manufacturer’s instructions. Seven serial 1/10 dilutions of each construct, with the highest concentration being 10 pg/μl of plasmid, were analysed invariably on every qPCR plate, for the purpose of generating a 7 points standard curve, from the slope of which a satisfactory 95–105 % efficiency of amplification was ascertained. The equation of the standard curve was used to ascribe a DNA quantity to each obtained Ct value, provided that the latter would not be higher than the Ct obtained from the most diluted standard sample, and from that quantity an absolute target copy number could be calculated. All samples were run in two technical replicates on every plate. Moreover, a dissociation curve was performed after every run, in order to assess whether the obtained fluorescence signals (particularly of high Ct values) corresponded to the desired product and not to accumulation of primer dimers or unspecific products. The cycling conditions, identical for all target sequences, were the following: 95 °C 2 min, (95 °C 10 s, 62 °C 30 s) × 40 cycles. For the melting curve, products were denatured at 95 °C for 1 min, allowed to re-anneal at 60 °C for 30 s, and then the temperature was gradually raised up to 95 °C, with data collection at every +0.3 °C increment. In order to express the relative enrichment of a specific histone mark over a particular genomic locus of interest, a double normalisation approach was employed (Morohashi et al. 2009), according to which the IP DNA quantity was first normalised against the corresponding Input DNA quantity (% of Input) and the obtained ratio was afterwards normalised against the similar ratio obtained with a reference primer set (either *ACTIN2* or *UBQ5*). Independent biological ChIP experiments were always performed with the same lot of extraction buffers and solutions, and, whenever possible, the recovered DNA was analysed with the same qPCR master mix, to reduce the risk of added variation, technical in its origin. Results are presented with standard deviation (SD) error bars, which emphasize variability and have been proposed to be more appropriate in reporting quantitative ChIP data (Struhl 2007). Two tailed Student’s *t* tests, resulting in p values, were employed to assess statistical significance between pairs of values, with the p value threshold representing the lowest acceptable confidence limits being set to p < 0.1 (Guo et al. 2008). Statistical analysis was performed with Wizard (Version 1.5.1 (101) available from Apple store).

For ChIP-Seq analysis the recovered DNA from 6 independent ChIP experiments was combined and sequencing was carried out in Glasgow Polyomics Facility (University of Glasgow). DNA libraries were prepared using the NEB DNA Ultra kit (New England BioLabs Inc.) according to the manufacturer’s protocol, size selected on an agarose gel, amplified by PCR and sequenced with the Illumina NextSeq 500 sequencer producing single 76 bp reads. For each sample the unaligned reads in fastq format were aligned to the *A. thaliana* genome (TAIR10) using Bowtie version 0.12.7 (Langmead et al. 2009) allowing for unique read alignments only with up to two mismatches in the first 54 bases. The alignment files in SAM/BAM format were sorted and duplicated reads with the same orientation removed using Samtools (Li et al. 2009) and converted to BED format. The differences in histone modification levels were analysed with ChIPDiff software (Xu et al. 2008) using default parameters except for the effective genome fraction set to 0.94 (Sani et al. 2013), window size set to 200 bp, fold-change threshold of 1.2, fragment sizes set to library specific values generated with Bioanalyzer and optimal internal gap length set to 200 bp after optimisation procedure run with Sicer software (Zang et al. 2009) as described in Sani et al. (2013).

### Acetylation inhibitor experiments

Three week old plants, grown on 70 mm diameter filter paper placed on top of 1/2 MS agar plates in a growth cabinet with 16 h light (60 µmol m^−2^ s^−1^)/8 h dark cycle, were either dark adapted for 16 h (gene expression experiments) or not (ChIP experiments) prior to treatment with inhibitors and UV-B illumination. Inhibitors were administered as follows: the filter paper with the plants was detached from the agar plates, rolled (plants facing inwards) and fitted in a 25 ml centrifuge tube which was then filled with a working concentration of either anacardic acid (57, 114 or 228 mM), or curcumin (100, 200, 300 or 400 μM), or DMSO (0.8 % v/v in water) as a solvent control. Samples were placed in a desiccator, subjected to vacuum infiltration for 15 min, and then the filter paper with the plants was reattached on the agar plates and tissue corresponding to 0 h UV-B control was harvested. Alternatively, the inhibitor-infiltrated plants were illuminated for 1 h with 1.5 μmol m^−2^ s^−1^ narrowband UV-B and either used for ChIP experiments, or covered in foil for an additional 2 h before harvesting for gene expression experiments (to allow maximum transcript accumulation). For ChIP experiments the harvested material was subjected to the formaldehyde fixation step before storing. For gene expression experiments the harvested material was washed with water, immediately snap frozen in liquid nitrogen and stored at −80 °C until used. ChIPs were carried out as already described and RT-qPCR analysis was conducted as by Livak and Schmittgen (2001).

## Results

### UVR8 affects the acetylation status of lysines K9 and K14 of Histone H3 on the chromatin of certain UVR8-regulated UV-B-responsive loci

**Fig. 1:**
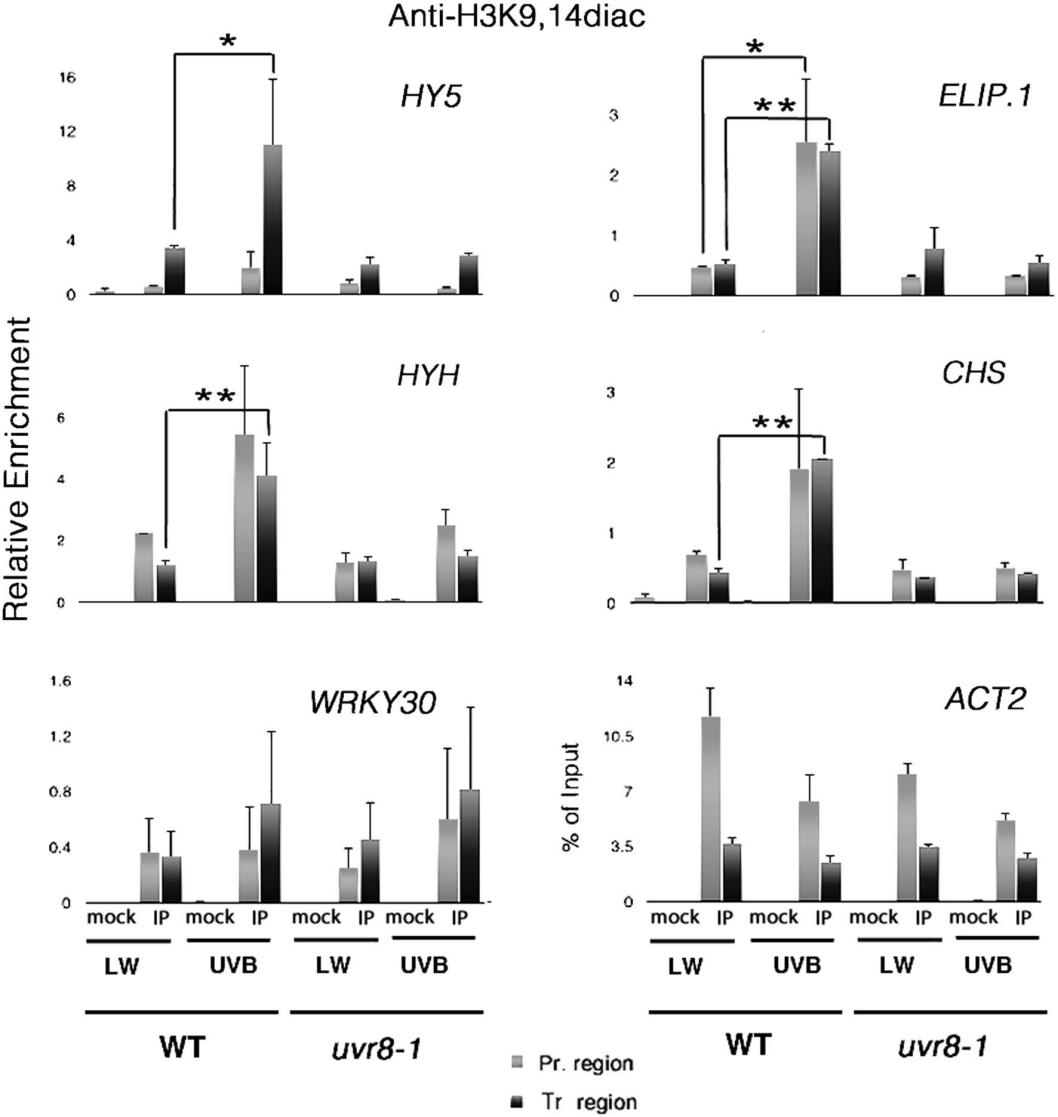
UVR8 regulates acetylation of lysines K9 and K14 of histone H3 on several UVR8-regulated genetic loci. Each graph displays the relative enrichment in H3K9,14diac for both wild-type (WT) and *uvr8-1* plants, on promoter (*light gray*) and transcribed regions (*dark gray*) of the designated genes. Plants were grown under low fluence rate white light (15 μmol m^−**2**^ s^−**1**^) with no measurable UV-B (LW) and then exposed to 1.5 μmol m^−**2**^ s^−**1**^ narrowband UV-B for 4 h (UV-B). *Mock* no Ab control, *IP* immunoprecipitated material. Results are expressed as % of Input normalised against *ACT2* (relative enrichment). For *ACT2* itself, no normalisation was performed and enrichment is given as % of Input. *Error bars* represent SD (n = 3). *p < 0.1; **p < 0.05

Cloix and Jenkins (2008) reported that H3K9,14diac might be involved in the regulation of transcription by UV-B radiation. However, since this study used only wild-type plants, no conclusions could be drawn as to whether UVR8 is involved. Hence, to test this, wild-type and *uvr8-1* mutant plants, grown for 3 weeks under low fluence rate white light (LW) were transferred to 1.5 μmol m^−2^ s^−1^ UV-B for 4 h. Chromatin was immunoprecipitated with an antibody recognising H3K9,14diac and control ChIP experiments were performed with an antibody against an invariant histone H3 domain (Supplementary Fig. S1) to demonstrate that the UV-B treatment did not cause ChIP-detectable changes in nucleosome occupancy, which if present would pose a problem for the interpretation of the data. Specific UVR8-dependent UV-B induced genes, *CHS, ELIP1, HY5* and *HYH*, were examined by qPCR analysis of the immunoprecipitated DNA. *ACT2* was used as a reference gene for normalisation of the amount of the ChIPed material and *WRKY30*, a gene induced through stress-related UVR8-independent UV-B responses, was chosen as a control to assess whether the threshold separating the photomorphogenic from the stressful stimulus was exceeded during the UV-B treatments. As shown in Fig. 1, wild-type plants exhibited a significant increase in H3K9,14diac enrichment after UV-B treatment over the transcribed regions of *HY5* (p = 0.08), *ELIP1* (p = 0.02), *HYH* (p = 0.04) and *CHS* (p = 0.02). No significant increase was seen for *uvr8-1* plants, and no significant difference was observed between wild-type and *uvr8-1* when *WRKY30* was assayed. In partial consonance with the findings of Cloix and Jenkins (2008), the promoter region of *ELIP1*, but not of *HY5, HYH* or *CHS*, was found to be significantly enriched in H3K9,14diac after UV-B exposure of the wild-type plants (p = 0.09). No such response was seen for the *uvr8-1* mutants. For the *HY5, HYH* and *CHS* promoters the p-values were found to be higher than the threshold of 0.1 (*HY5* p = 0.17, *HYH* p = 0.14, *CHS* p = 0.2). The threshold of p < 0.1 is not common in scientific practice, but because of the inherent variability of ChIP assays, it has been used to claim significance for quantitative ChIP results with the lowest acceptable confidence limits (Guo et al. 2008). Nevertheless, H3K9,14diac levels over the *HY5, HYH* and *CHS* promoters were consistently higher in UV-B-irradiated wild-type plants compared to controls, but not in *uvr8-1* plants. It is therefore possible that variation between individual experiments may have masked a mild but biologically important difference, which would only appear as statistically significant after many repetitions.

Collectively, the results indicate that UVR8 is required for the UV-B-induced accumulation of H3K9,14diac over the assayed genetic loci, and imply a novel mechanism of action of UVR8 during photomorphogenic UV-B responses.

### HY5 and/or HYH are required, at least for specific loci, for the UVR8-mediated UV-B-induced enrichment in H3K9,14diac

**Fig. 2:**
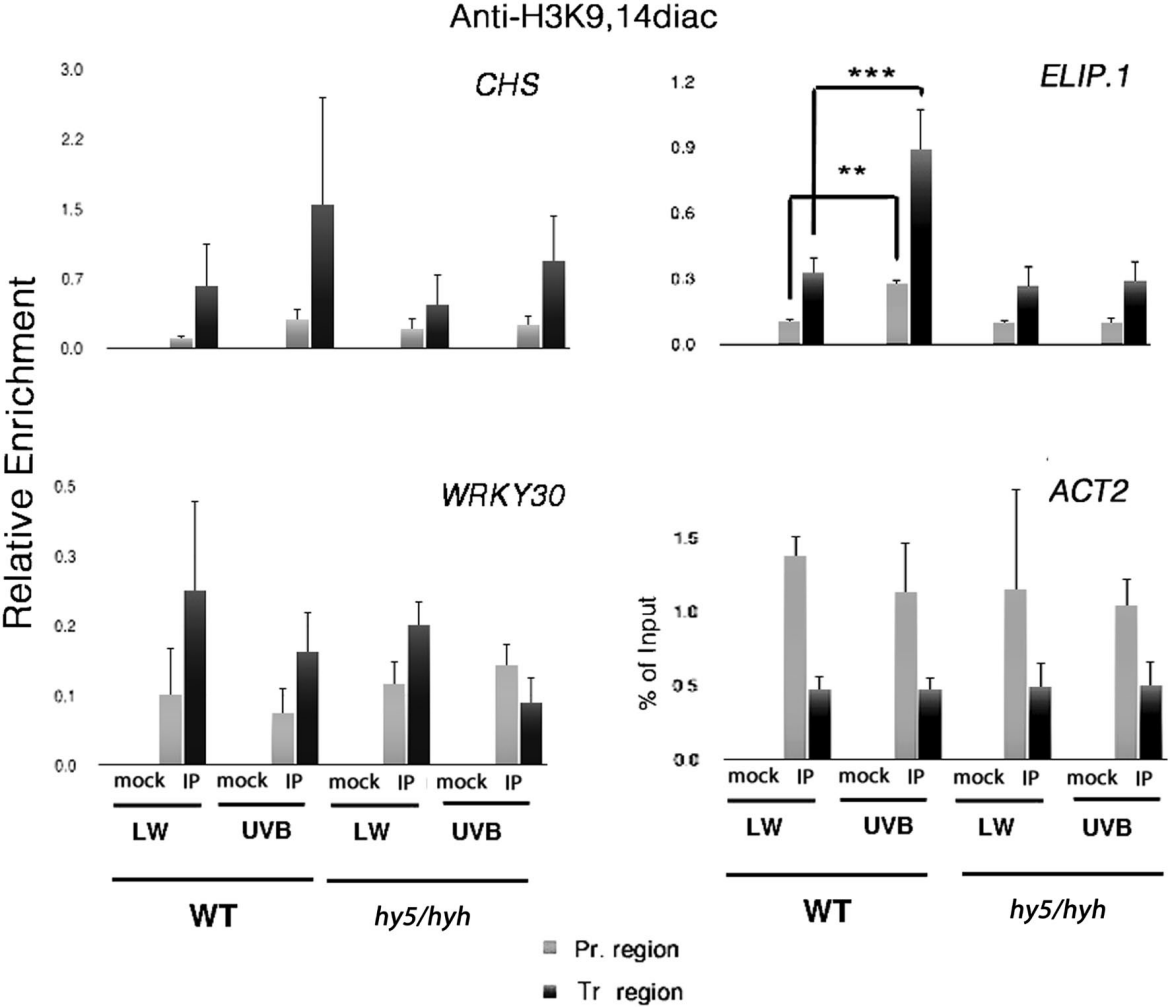
HY5 and/or HYH affect the acetylation status of lysines K9 and K14 of histone H3 on particular UVR8-regulated genetic loci. Each graph displays the relative enrichment in H3K9,14diac for both wild-type (WT) and *hy5*/*hyh* plants, on promoter (*light gray*) and transcribed regions (*dark gray*) of the designated genes. Plants were grown under low fluence rate white light (15 μmol m^−**2**^ s^−**1**^) with no measurable UV-B (LW) and then exposed to 1.5 μmol m^−**2**^ s^−**1**^ narrowband UV-B for 4 h (UV-B). *Mock* no Ab control. *IP* immunoprecipitated material. Results are expressed as % of Input normalised against *ACT2* (relative enrichment). For *ACT2* itself, no normalisation was performed and enrichment is given as % of Input. *Error bars* represent SD (n = 3). **p < 0.05; ***p < 0.01

The above observations raised the question of whether UVR8 itself is directly, physically involved in the accumulation of H3K9,14diac at specific loci or whether a downstream effector undertakes that role instead. A possible candidate would be HY5, which has been reported to be involved in the regulation of H3K9ac levels (Guo et al. 2008), and has been proposed to mediate the transcription of downstream targets synergistically with HYH and histone acetylation (Benhamed et al. 2008; Charron et al. 2009). To address this question we performed ChIP assays using wild-type and *hy5hyh* mutant lines. Interestingly, the qPCR analysis of the ChIPed material from these experiments revealed both similarities and differences compared to the results with *uvr8-1* plants. In particular (Fig. 2), both the promoter and transcribed regions of *ELIP1* were found significantly enriched in H3K9,14diac (*proELIP1* p = 0.03, *trELIP1* p = 0.0001) following UV-B treatment of wild-type plants, but no change was seen for *hy5hyh* double mutants. However, wild-type plants did not show a significant increase of acetylation levels over *CHS* following UV-B illumination. Although in principle this could be due to the use of wild-type Ws (the appropriate control for *hy5*/*hyh*), it is more likely the result of intrinsic variability among independent experiments. When the three independent repeats are viewed separately (Supplementary Fig. S2), it is apparent that, at least for the transcribed region of *CHS, hy5*/*hyh* shows a much reduced enrichment following UV-B exposure compared to wild-type. With regard to the control genes *ACT2* and *WRKY30*, wild-type and *hy5*/*hyh* plants showed similar acetylation patterns regardless of the illumination conditions. These findings imply that under UV-B, at least a subset of the UVR8-regulated genes undergo a HY5- and/or HYH- dependent accumulation of the H3K9,14diac histone mark.

### ChIPseq revealed that all UV-B-induced enrichments in H3K9,14diac observed across the genome are UVR8-dependent and more than one-third of the loci consist of known UVR8-regulated genes

**Fig. 3:**
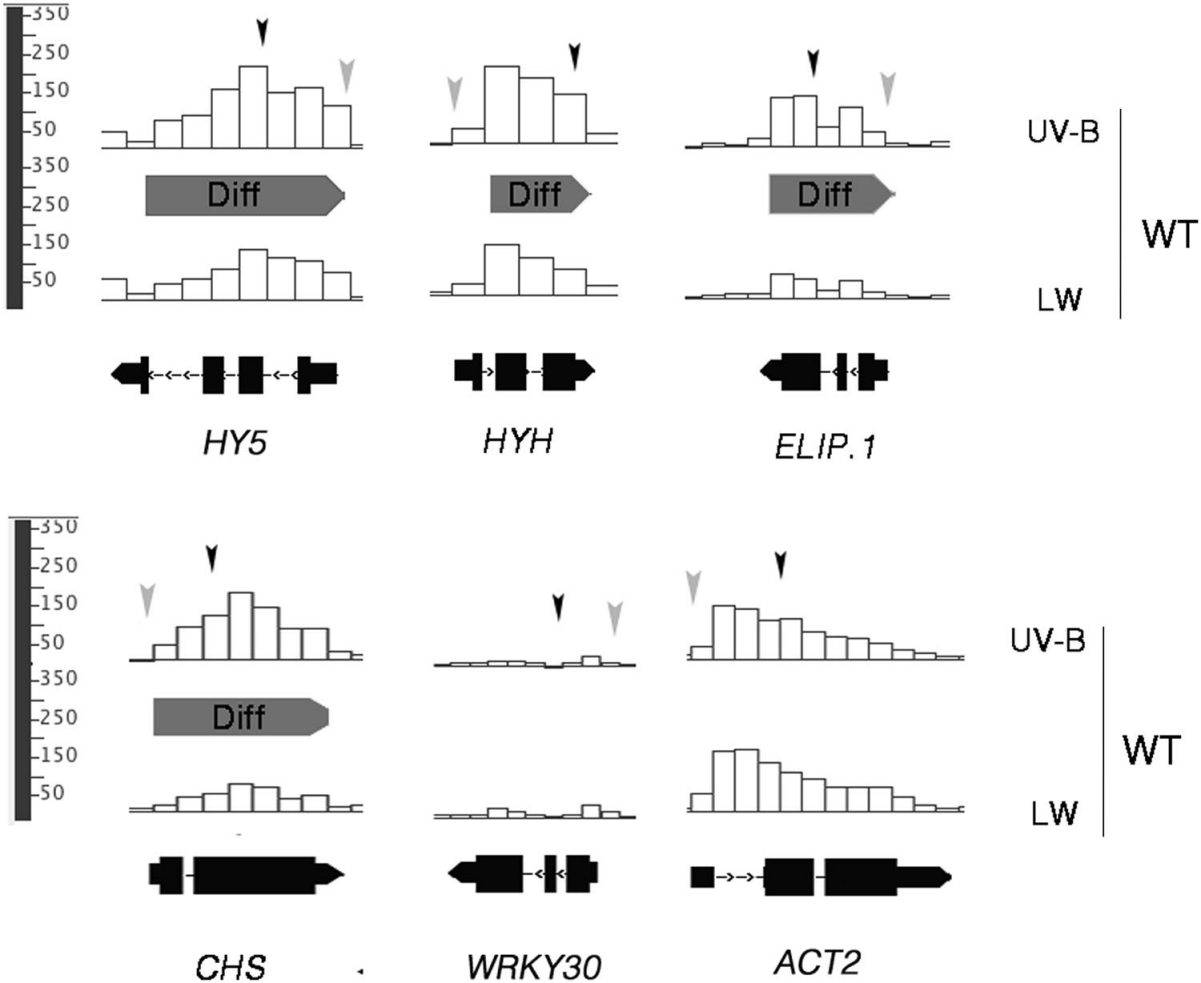
ChIPseq confirmed that *HY5, HYH, ELIP1* and *CHS* were among the genomic loci differentially enriched in H3K9,14diac after UV-B illumination. Snapshots from the Integrated Genome Browser (IGB) showing the relative positions of UV-B-induced differential sites (*gray* areas labeled Diff), as identified by the ChIPDiff software, within the genomic loci of *HY5, HYH, ELIP1* and *CHS* compared to the control genes *ACT2* and *WRKY30*. The *arrowheads* indicate the approximate positions of the qPCR amplicons in Fig. 1. *Grey arrowheads* promoter region; *black arrowheads* transcribed region. The results presented are for wild-type (WT) plants grown under low fluence rate white light (15 μmol m^−2^ s^−1^) with no measurable UV-B (LW), then exposed to 1.5 μmol m^−2^ s^−1^ narrowband UV-B for 4 h (UV-B)

**Fig. 4:**
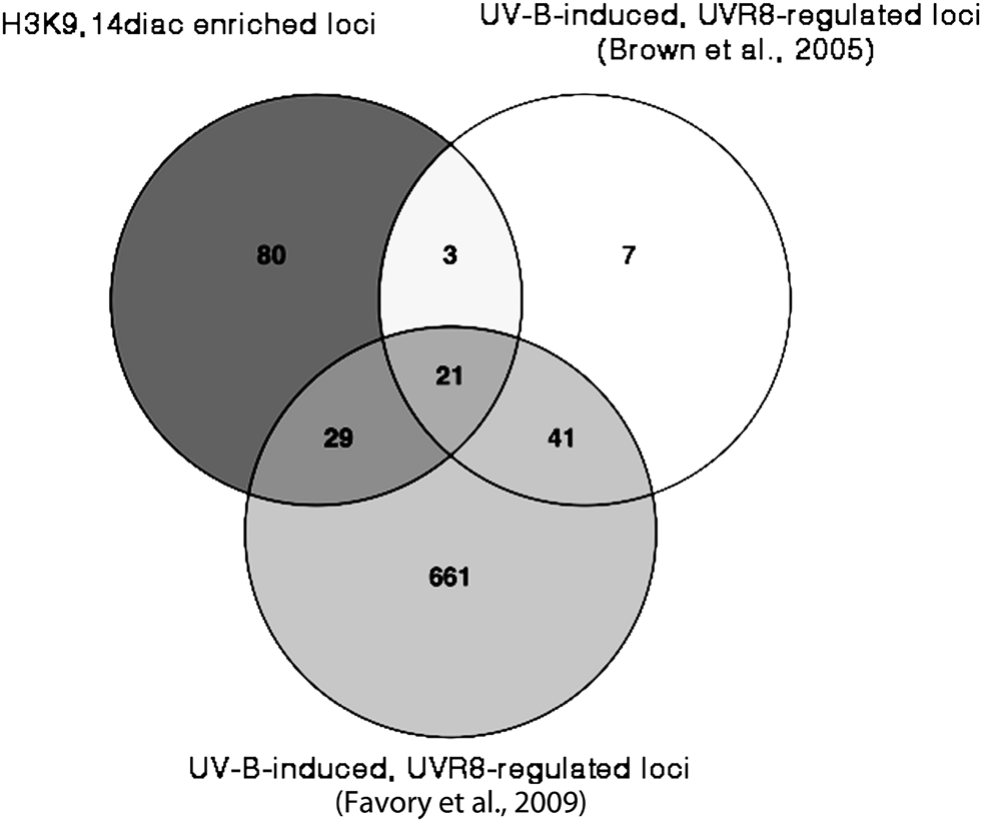
Substantial overlap between the set of genetic loci showing UVR8-dependent H3K9,14diac enrichment and published datasets of UVB-induced-UVR8-regulated genes. Venn diagram showing the extent of commonality between the 133 loci identified in this study to undergo UVR8-dependent accumulation of H3K9,14diac following UV-B illumination and genes that were reported to be up-regulated by UV-B under UVR8 regulation by either Brown et al. (2005) or Favory et al. (2009)

Targeted qPCR analysis of immunoprecipitated DNA is limited by the number of genes that can be examined. Hence, to identify, on a genome wide scale, loci which display UV-B induced, UVR8-dependent H3K9,14diac enrichment we performed ChIPseq. The set of genes identified by this analysis was compared with datasets derived from microarray experiments which have highlighted genes that are UVR8-regulated, or HY5-regulated, or UV-B regulated in general. In order to identify genomic regions that differed in histone acetylation we used the ChIPDiff software (Xu et al. 2008). Only the sites whose enrichment levels appear significantly larger than those in the neighbouring regions are extracted and the overall noise is subtracted from the profiles. Comparison of the acetylation sites of UV-B-illuminated wild-type plants with those kept under control light conditions identified 140 differential positions, the vast majority of which (133 sites) represented enrichments (Supplementary Table S3). The relevant changes were small, occurring only at the minimal cut-off threshold of 1.2-fold. Nevertheless, such differences have been reported to be biologically meaningful in other works (Sani et al. 2013) and could, in principle, be an underestimation of the true *in planta* enrichments for some loci. The immunoprecipitated material recovered from independent biological replications of ChIP experiments is often pooled in one combined sample prior to sequencing, thereby averaging potentially outlying values. It is imperative, therefore, to confirm ChIPseq results for certain genetic loci of interest. Importantly, we determined that *HY5, HYH, CHS* and *ELIP1*, all previously identified via targeted qPCR analysis as undergoing UVR8-dependent H3K9,14diac accumulation upon UV-B illumination, appear in the list of the differentially enriched genomic regions (Table 1; Fig. 3). Furthermore, no locus in our entire dataset had dissimilar H3K9,14diac levels when the two light conditions were compared for *uvr8-1* plants, supporting the conclusion that UV-B-induced enrichments in H3K9,14diac require the presence of a functional UVR8. When the dataset was compared with published microarray data, there was a 17 % overlap with the UVB-induced, UVR8-regulated genes published by Brown et al. (2005), who also used 3 week-old plants, and 37 % overlap with the more extensive dataset of Favory et al. (2009) who analysed 4 day-old seedlings (Fig. 4). Taking both studies into account, it appears that 39 % of the genomic sites we identified as displaying a UVR8-dependent H3K9,14diac accumulation following UV-B illumination correspond to genes whose transcripts are regulated by UVR8. A low percentage, only 9 %, of our dataset was found to overlap with a list of UV-B-induced, HY5-regulated genes (Brown and Jenkins 2008; Oravecz et al. 2006). However, these studies used *hy5-1* plants, which have a functional HYH protein, and so the results are influenced by the documented partial functional redundancy of these transcription factors.

**Table 1:** List of known UV-B-induced UVR8-regulated genes (Brown et al. 2005; Favory et al. 2009) that were detected to undergo UV-B induced, UVR-8-dependent H3K9,14diac enrichment in this study

Chromosome	TAIR annotation	Name	Description	Chromosome	TAIR annotation	Name	Description
Chr1	AT1G01520		Homeodomain-like superfamily protein	Chr3	AT3G51240	F3′H|F3H|TT6	Flavanone 3-hydroxylase
Chr1	AT1G02340	RSF1|REP1|HFR1|FBI1	Basic helix-loop-helix (bHLH) DNA-binding superfamily protein	Chr3	AT3G52740		Unknown protein
Chr1	AT1G02820		Late embryogenesis abundant 3 (LEA3) family protein	Chr3	AT3G56290		Unknown
Chr1	AT1G06430		FTSH protease 8	Chr3	AT3G57020		Calcium-dependent phosphotriesterase superfamily protein
Chr1	AT1G12370	PHR1|UVR2	Photolyase 1	Chr3	AT3G57520	SIP2|AtSIP2	Seed imbibition 2
Chr1	AT1G17050		Solanesyl diphosphate synthase 2	Chr3	AT3G61220		NAD(P)-binding Rossmann-fold superfamily protein
Chr1	AT1G23550		Similar to RCD one 2	Ch4	AT4G00050		Basic helix-loop-helix (bHLH) DNA-binding superfamily protein
Chr1	AT1G64500		Glutaredoxin family protein	Ch4	AT4G05100		myb Domain protein 74
Chr1	AT1G79270	ECT8	Evolutionarily conserved C-terminal region 8	Ch4	AT4G14690	ELIP2	Chlorophyll A–B binding family protein
Chr2	AT2G15020		Unknown protein	Ch4	AT4G16690	MES16|ATMES16	Methyl esterase 16
Chr2	AT2G16365		F-box family protein	Ch4	AT4G27030	FADA|FAD4	Fatty acid desaturase A
Chr2	AT2G21970		Stress enhanced protein 2	Ch4	AT4G31870	GPX7|ATGPX7	Glutathione peroxidase 7
Chr2	AT2G24540	AFR	Galactose oxidase/kelch repeat superfamily protein	Ch4	AT4G37150	MES9|ATMES9	Methyl esterase 9
Chr2	AT2G25450		2-Oxoglutarate (2OG) and Fe(II)-dependent oxygenase superfamily protein	Ch4	AT4G37760	SQE3	Squalene epoxidase 3
Chr2	AT2G29460	GST22|GSTU4|ATGSTU4	Glutathione S-transferase tau 4	Ch5	AT5G11260	HY5	Basic-leucine zipper (bZIP) transcription factor family protein
Chr2	AT2G37970		SOUL heme-binding family protein	Ch5	AT5G13930	CHS	Chalcone and stilbene synthase family protein
Chr2	AT2G40460		Major facilitator superfamily protein	Ch5	AT5G17780		Alpha/beta-Hydrolases superfamily protein
Chr3	AT3G10910		RING/U-box superfamily protein	Ch5	AT5G19850		Alpha/beta-Hydrolases superfamily protein
Chr3	AT3G14770		Nodulin MtN3 family protein	Ch5	AT5G23730	RUP2	Transducin/WD40 repeat-like superfamily protein
Chr3	AT3G17609	HYH	HY5-homologue	Ch5	AT5G24120		Sigma factor E
Chr3	AT3G21560	UGT84A2	UDP-glycosyltransferase superfamily protein	Ch5	AT5G24150		FAD/NAD(P)-binding oxidoreductase family protein
Chr3	AT3G21890		B-box like zinc finger protein	Ch5	AT5G37550		Unknown protein
Chr3	AT3G22840	ELIP1|ELIP	Chlorophyll A-B binding family protein	Ch5	AT5G42760		Leucine carboxyl methyltransferase
Chr3	AT3G24170	ATGR1|GR1	Glutathione-disulfide reductase	Ch5	AT5G53970		Tyrosine transaminase family protein
Chr3	AT3G27170		CLC-B|ATCLC-B	Ch5	AT5G55570		Unknown protein
Chr3	AT3G48460		GDSL-like lipase/acylhydrolase superfamily protein	Ch5	AT5G58760	DDB2	Damaged DNA binding 2
				Ch5	AT5G58770		Undecaprenyl pyrophosphate synthetase family protein

**Fig. 5:**
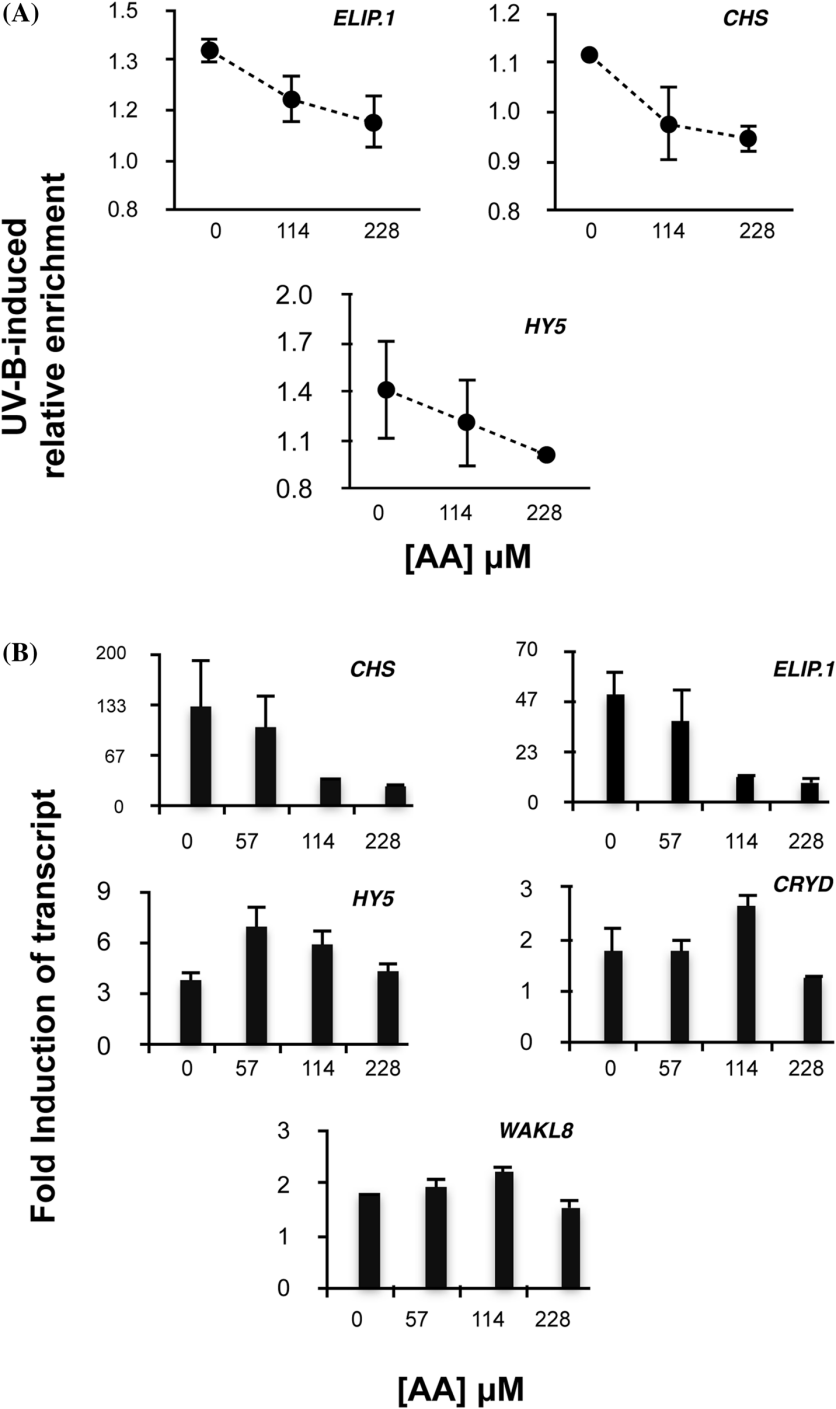
Anacardic acid inhibits UV-B induced H3K9,14diac enrichment and expression of specific UVR8-regulated genes. **A** Relative UV-B induced enrichment of H3K9,14diac at selected loci, assayed by ChIP-qPCR, and **B** fold UV-B induction of transcripts of selected genes, measured by RT-qPCR, in plants treated with increasing concentrations of anacardic acid (AA). Plants were infiltrated with the inhibitor for 15 min and then exposed (or not in controls) to 1.5 μmol m^−2^ s^−1^ narrowband UV-B for 1 h. Plants were harvested immediately for ChIP assays or harvested after 2 h in darkness for RT-qPCR. *Error bars* represent SD (n = 3)

**Fig. 6:**
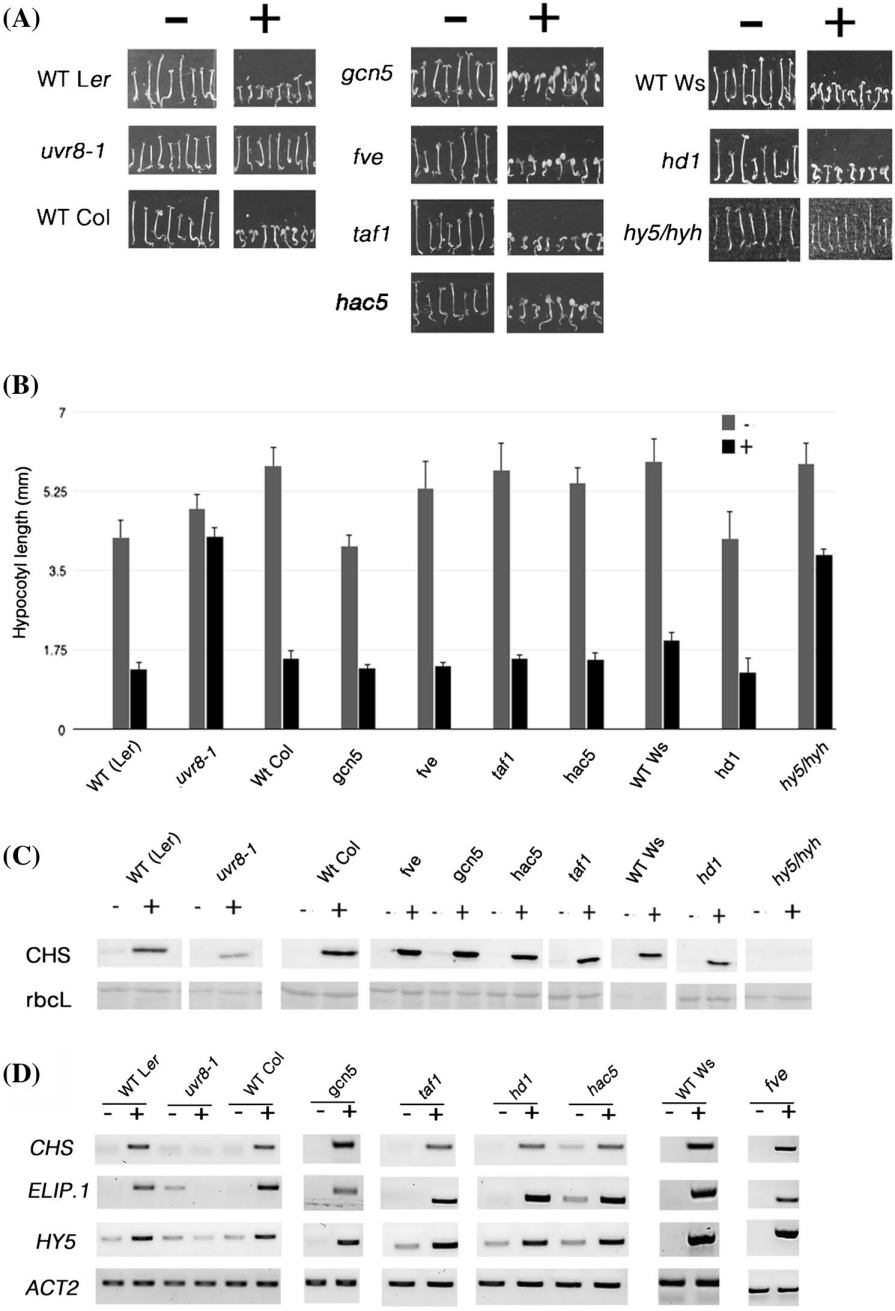
UVR8 mediated responses are unaltered in selected HAT/HDAC mutants. **a** Photographs of representative seedlings from each line, germinated and grown for 5 days in low fluence rate white light (1.5 μmol m^−2^ s^−1^) supplemented (+) or not (−) with 1.5 μmol m^−2^ s^−1^ narrowband UV-B. **b** Average hypocotyl length of seedlings shown in (**a**). *Error bars* represent SE (n ≥ 25). **c** Anti-CHS antibody immunoblots of protein samples prepared from crude protein extracts of 5 days-old seedlings grown as in (**a)**. Ponceau staining of the RuBisCo large subunit (rbcL) is shown as a loading control. **d** Semi-quantitative RT-PCR showing transcripts of three UVR8 regulated genes compared to *ACT2* control transcripts in plants exposed (+) or not (−) to 1.5 μmol m^−2^ s^−1^ narrowband UV-B or control 15 μmol m^−2^ s^−1^ white light for 3 h. For **c** and **d** results are representative of three independent repeats

**Fig. 7:**
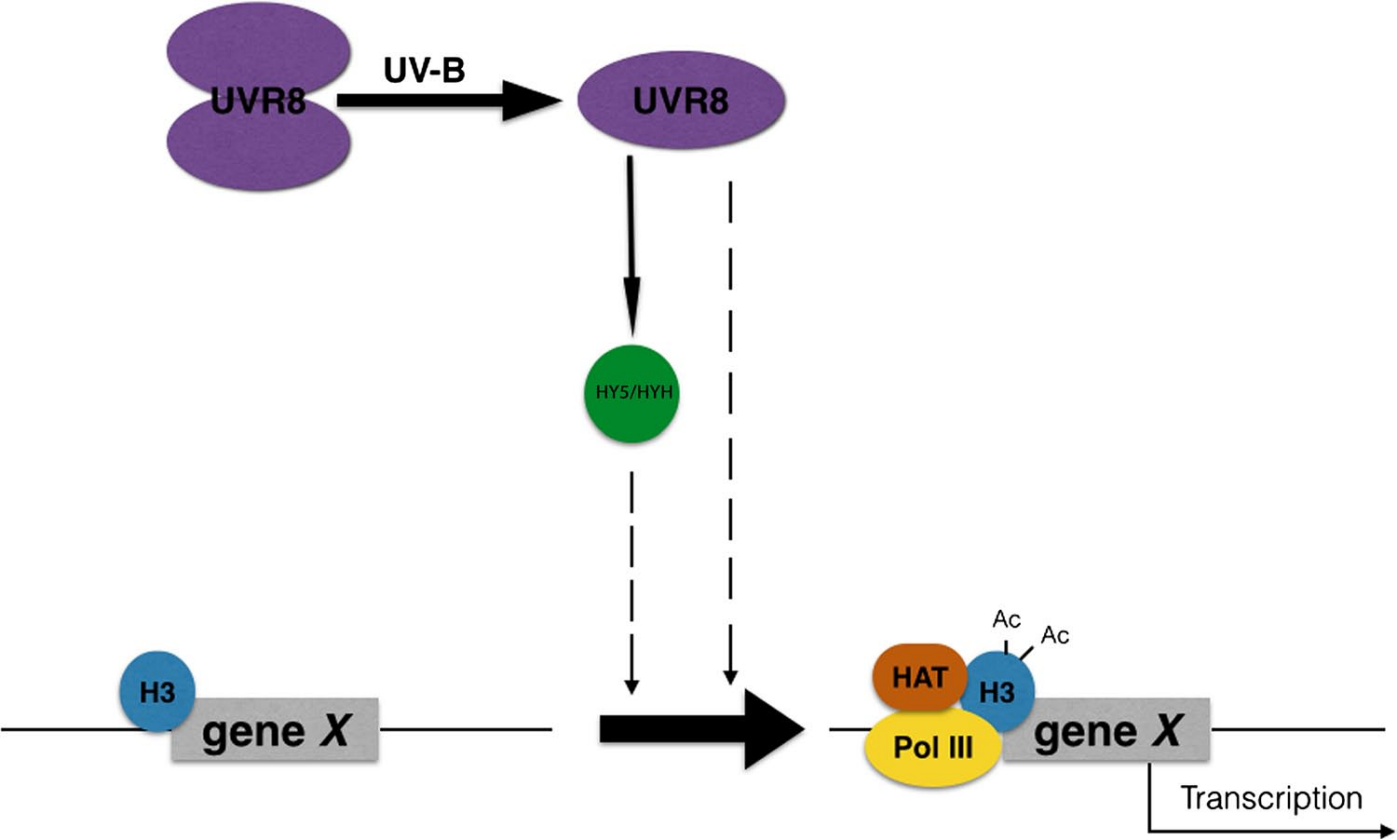
Model of UVR8 regulation of transcription. Photoreception of UV-B by dimeric UVR8 initiates monomerisation. UVR8 regulates the accumulation of HY5 and HYH transcription factors. UVR8 stimulates H3K9,14diacetylation at genomic loci containing UVR8-target genes (represented by gene X), promoting their transcription. HY5/HYH are also required for H3K9,14 diacetylation of at least some UVR8-target gene loci. The *dashed arrowed lines* indicate that it is unclear whether UVR8 and HY5/HYH recruit histone acetylation proteins through direct association with a multi-protein complex on chromatin or whether they stimulate histone acetylation indirectly

### UVR8 has no effect on the accumulation of H3K4me3, H2Bub, H3K9me3 or H3K36me3 but may be linked to a locus-specific accumulation of H3K56ac

We investigated whether UVR8 regulates the presence of other histone modifications. Publications on genome-wide epigenetic mapping have revealed that certain histone modifications tend to co-appear in some epigenetic landscapes, whereas others tend to be mutually exclusive (Charron et al. 2009; Roudier et al. 2011; Zhang et al. 2009). On this basis we selected five histone modifications for analysis, namely H3K4me3, H2Bub, H3K9me3, H3K36me3 and H3K56ac. After performing control experiments to establish that non-target chromatin is not recovered in the immunoprecipitated material (Supplementary Fig. S3), we undertook ChIPs with essentially the same experimental set up as for H3K9,14diac. Our results, summarised in Table 2 and presented in Supplementary Fig. S4 to Fig. S8, revealed no significant effect of UV-B on the accumulation of the tested histone marks when the two distinct light conditions were compared within each genotype. Nevertheless an intriguing pattern was detected for H3K56ac (Supplementary Fig. S8). In particular, for both *ELIP1* and *CHS*, wild-type plants displayed significantly higher H3K56ac enrichment levels over the transcribed region compared to *uvr8-1* plants, when the cumulative signals for the two light conditions were examined (*ELIP1* p = 0.005; *CHS* p = 0.008). In addition, a two-way ANOVA suggested that the combined influence of UVR8 loss-of-function and UV-B illumination has a cumulative effect in decreasing the average relative enrichment levels of H3K56ac over the transcribed region of *ELIP1* (p = 0.05, 94 % Confidence). Although the data are based on only two independent biological replicates, the observations suggest that a functional UVR8 is required for keeping adequate levels of H3K56ac over these loci. No equivalent pattern was observed for *HY5* and *HYH*. Thus, for at least a subset of the UVR8-regulated genes, H3K56ac could be involved in the increased gene expression that follows UV-B illumination, and the deposition and/or removal of this histone mark might be linked to UVR8.

**Table 2:** Synopsis of the ChIP-qPCR results obtained in this study

	H3K9,14diac	H2Bub	H3K4me3	H3K9me3	H3K36me3	H3K56ac
*HY5*	++	−	−	−	−	−
*HYH*	++	−	−	−	−	−
*ELIP1*	++	−	−	−	−	+
*CHS*	++	−	−	−	−	+
*WRKY30*	−	−	−	−	−	−
*ACT2*	−	−	−	−	−	−

The table summarises the observations from the ChIP experiments performed on WT and *uvr8-1* plants during this study. The plus sign (+) highlights those occasions on which different enrichment patterns were observed between the two genotypes, whereas the minus sign (−) is used to indicate that no detectable differences were observed. The number of + signs (2 vs. 1) was used to denote differences in the statistical rigour of the observations. The results for H4K56ac were obtained from two independent biological replicates, in contrast to H3K9,14diac, for which 3 independent biological replicates were assayed and the results were further confirmed, independently, by the ChIPseq

### Inhibition of histone acetylation impairs UVR8 mediated induction of *ELIP1* and *CHS* expression

To obtain further insights into the involvement of histone acetylation in UVR8 mediated transcription, we employed anacardic acid (AA), a potent generic inhibitor of p300/CBP and PCAF histone acetyltransferase activities (Balasubramanyam et al. 2003). AA administration led to a concentration dependent attenuation of UV-B-induced H3K9,14diac enrichment at genetic loci of interest (Fig. 5a) and a concomitant inhibition of UV-B induced transcript accumulation of the UVR8 regulated genes *CHS* and *ELIP1* (Fig. 5b) Interestingly, AA had a lesser effect on the increase in *HY5* transcripts. *CRY3 (AT5G24850)* and *WAKL8 (AT1G16260)* were included in the analysis as controls; both are known UVR8-regulated genes but neither was found enriched in H3K9,14diac in our ChIPseq dataset and were therefore predicted to remain unaffected by the inhibitor. When curcumin (Cur), a slightly more specific inhibitor that does not target PCAFs (Balasubramanyam et al. 2004), was used instead of AA, the results were more variable (Supplementary Fig. S9) and high concentrations had to be used (fourfold more than active concentrations reported previously; Casati et al. 2008).

### GCN5, TAF1, HAC5, HD1 and FVE are not required for several UVR8-dependent photomorphogenic UV-B responses

Since our findings implicated the acetylation status of specific histone H3 residues in the transcriptional regulation of photomorphogenic UV-B responses mediated by UVR8, we investigated whether particular histone acetyltransferases (HATs) and/or histone de-acetylases (HDACs) were involved. It is conceivable that UVR8 could be directly involved in the recruitment of the relevant histone modifying enzymes. We therefore used yeast two hybrid (Y2H) assays to test interactions between UVR8 and candidate HATs/HDACs with documented involvement in light signalling. We also examined proteins that have no histone modifying activity themselves, but whose presence facilitates HAT/HDAC activity, and several additional candidates for direct interaction with UVR8 in a chromatin context based on relevant publications (Ausín et al. 2004; Barneche et al. 2014; Benhamed et al. 2006, 2008; Bertrand et al. 2005; Campi et al. 2012; Fisher and Franklin 2011; Pazhouhandeh et al. 2011).

The HAT General Control Nonderepressible protein 5 (GCN5) has been implicated in light-inducible gene expression and is required for H3K9ac and H3K14ac modifications (Benhamed et al. 2006, 2008). However, neither GCN5 nor the functionally associated ADA2 (Alterations/Deficiency in Activation) members interacted with UVR8 in yeast (Supplementary Fig. S10). The Arabidopsis TATA Binding Protein (TBP)—Associated Factor 1 (TAF1) possesses HAT activity and is required for H3K9ac modification, acting together with *GCN5* (Benhamed et al. 2006). TAF1 is involved in light-regulated transcription through synergistic effects with HY5 (Bertrand et al. 2005). In contrast, HD1 (Histone Deacetylase 1, also known as HDA19), a member of the Arabidopsis RPD3 family of HDACs, is proposed to act antagonistically to GCN5 in regulating H3K9ac levels on the promoters of various light regulated genes (Benhamed et al. 2006). It has been suggested that HD1 might be involved in the maintenance of H3K9ac levels in a light-dependent manner (Guo et al. 2008). No interaction of UVR8 with either TAF1 or HD1 could be detected (Supplementary Fig. S11), nor with HAC5 (Histone Acetyltransferase 5), which has been reported to be capable of acetylating either lysine K9 or K14 of histone H3 (Earley et al. 2007).

In Arabidopsis, Damaged DNA Binding Protein 1 (DDB1) associates in a nucleosomal context with De-etiolated 1 (DET1) to repress transcription (Schroeder et al. 2002; Benvenuto et al. 2002). Upon illumination, DDB1 is thought to recruit HATs that acetylate the N-terminal tail of H2B, thereby leading to transcriptional up-regulation. DDB1 was found to interact with the WD40 domain of Flowering Locus VE (FVE) protein, which promotes flowering through histone deacetylation (Ausín et al. 2004) and chromatin remodelling (Pazhouhandeh et al. 2011). The two Arabidopsis homologues DDB1a and DDB1b, together with DDB2, constitute the DDB complex, which appears to be important for UV-B tolerance and genomic integrity (Biedermann and Hellmann 2010; Koga et al. 2006; Molinier et al. 2008). Intriguingly, *fve* mutant plants were recently reported to have reduced levels of histone acetylation following UV-B treatment, and accumulated cyclobutane pyrimidine dimers (Campi et al. 2012). No interaction was observed between UVR8 and either DDB1a, DDB1b or DDB2 (Supplementary Fig. S12, Fig. S13), whereas a weak interaction between UVR8 and FVE was detected when yeast cells were not exposed to UV-B (Supplementary Fig. S12).

Since we obtained evidence that HY5/HYH are required for enrichment of H3K9,14diac at the *ELIP1* locus, we tested whether HY5 might be involved in recruiting candidate histone modifying enzymes using the yeast 2-hybrid assay. However, no evidence was obtained for interaction of HY5 either with UVR8 or with the histone modifying enzymes tested (Supplementary Fig. S14).

*In planta* functional analyses were undertaken for the HATs and HDACs of interest. SALK-identified T-DNA insertional mutant lines were obtained and confirmed by PCR-based genotyping and RT-PCR (Supplementary Fig. S15) and several UVR8-mediated photomorphogenic UV-B responses were examined. Firstly, in the suppression of hypocotyl elongation by UV-B, all mutant lines of interest appeared to be UV-B responsive, exhibiting at least threefold shorter hypocotyls under UV-B, similar to their wild-type counterparts and in contrast to *uvr8-1* and *hy5*/*hyh* plants (Fig. 6). In addition, CHS protein levels, and transcript levels of three UVR8-regulated genes were markedly increased upon UV-B illumination in all mutant lines of interest, similar to wild-type plants (Fig. 6).

Taken together, our data imply that none of the HATs/HDACs of interest is essential for the UV-B responses that we tested. We conclude that either they are not involved at all, or they have a dispensable role which is fulfilled, in their absence, by a functionally related histone modifying protein.

## Discussion

Exploration of the in vivo function and mechanism of action of UVR8 is still at an early stage. Research in recent years has provided insights into the UVR8 photoreception mechanism, signalling initiation, regulation, and its physiological functions (Jenkins 2014a, b; Tilbrook et al. 2013), but there is a substantial gap of knowledge regarding the mechanism of UVR8-regulated transcription. The significance of the association of UVR8 with chromatin remains an enigma; it is not clear whether the detection by ChIP of UVR8 at some loci it regulates (Brown et al. 2005; Cloix and Jenkins 2008) indicates a functional necessity for the recruitment and/or activation of chromatin modifiers and/or transcription factors, which would underpin transcriptional regulation, or whether the association is simply due to non-specific chromatin binding because of an affinity for histones (Brown et al. 2005; Cloix and Jenkins 2008). UVR8’s association with chromatin is certainly much weaker than that of transcription factors such as HY5, raising questions regarding its significance (Binkert et al. 2016). The main objectives of this study were to test whether UVR8 influenced transcription through chromatin modification and to investigate whether it interacted directly with several known chromatin modifiers.

Alterations in the methylation status of DNA, rearrangements of the positions of nucleosomes and changes in the covalent modifications of protruding histone tails, are all common phenomena which ensure that the chromosomal DNA will remain in a loosely packed euchromatic state, thus facilitating the access, assembly and function of the transcriptional machinery on target genetic loci (Clapier and Cairns 2009; Ito et al. 2015; Jarillo et al. 2009; Li et al. 2007). We principally focused on the histone modifications facet of chromatin plasticity, because prior research had already provided some interesting initial findings (Casati et al. 2008; Cloix and Jenkins 2008). These studies had reported the potential significance of H3K9/14diac in UV-B responses but were restricted to a few genes and no links with UVR8 had been established. Our results extend these previous studies and provide evidence that the UV-B-induced enrichment in H3K9/14diac requires a functional UVR8 photoreceptor (Figs. 1, 3 and ChIPseq results). Chromatin loci occupied by genes encoding key proteins in photomorphogenic UV-B responses, such as HY5, HYH and CHS were found to undergo a UVR8-dependent accumulation of the above histone modification following UV-B illumination. Furthermore, sequencing of the immunoprecipitates revealed the genome-wide significance of the enrichment; 133 loci (Table 1 and Supplementary Table S3) displayed a UV-B-induced UVR8-dependent increase in acetylation and a substantial proportion (39 %, Fig. 3) corresponded to known UVR8-regulated genes. Notable examples, besides those already mentioned, include the transcription factor genes *HFR1, MYB74, SIG5*, the protease and photolyase genes *FTSH8* and *PHR1*/*UVR2*, the gene encoding the negative regulator of photomorphogenic UV-B responses RUP2, and Supplementary Table S3. The 39 % overlap of the differentially enriched loci with known UVR8-regulated genes is much higher than the percentage one would reasonably expect by pure chance. Moreover, the complete lack of differential sites when the two light conditions were compared for *uvr8-1* plants strengthens the notion that UVR8 regulates a specific chromatin modification that is associated with transcriptional regulation of a set of target genes. Nevertheless, it is evident that many genes identified in the microarray analyses were not detected by ChIP sequencing. This may in part be explained by technical factors associated with the methods, which limit comparison, but it is important to note that while enrichment of H3K9/14diac may strongly influence the rate of transcription, the latter is not solely dependent on acetylation; additional factors, including other chromatin modifications, may also be involved.

At this point it should be noted that antibodies raised against bivalent immunogens may have an undocumented preference over one of the two modifications (Perez-Burgos et al. 2004), and caution is therefore advisable when reaching conclusions. Recently, Schenke et al. (2014) used antibodies raised specifically against H3K9ac or H3K14ac, and by employing UV-B illumination conditions that are just sufficient to trigger photomorphogenic responses concluded that the UV-B-associated increases in H3 acetylation at particular gene loci are a consequence of H3K9ac. Therefore, it is likely that our observations are also an effect of H3K9ac rather than H3K14ac, although clearly for this claim to be fully justified the specific antibodies must be used.

The above findings are consistent with the concept of a functionally relevant UVR8-chromatin association. However, the direct association of UVR8 with a protein complex on chromatin is not necessarily essential to initiate H3K9/14diac accumulation at target loci. One might argue that in *uvr8-1* mutants the whole downstream photomorphogenic UV-B signalling pathway is not operational, and hence it is not the physical absence of UVR8 itself that causes the observed results but, rather, a downstream effector has not been diverted towards mediating the enrichments. HY5 has been suggested as capable of fulfilling such a role (Barneche et al. 2014; Charron et al. 2009; Guo et al. 2008), and our analysis revealed that indeed, at least for selected UVR8-regulated loci, HY5 and/or HYH are involved in UV-B-induced H3K9/14diac accumulation (Fig. 2 and Fig. S2). *HY5* and *HYH* have not been presented in the corresponding ChIP-qPCR results because the T-DNA insertions in *hy5*/*hyh* plants disrupt precisely those two loci and the DNA sequence in these positions is no longer comparable to that of the wild-type. A worthwhile future research effort would be to conduct ChIPseq experiments with *hy5*/*hyh* plants and then to compare the resulting dataset of differentially enriched loci with the one reported herein. This could also help to resolve puzzling observations made for *CCA1*; the *CCA1* promoter has been reported to bind HY5 (Lee et al. 2007) and was found in our experiments to accumulate H3K9/14diac upon UV-B exposure (Supplementary Table S3), but the gene itself is reported to be UVR8-regulated in a HY5-independent manner (Feher et al. 2011).

It is interesting that for those genes examined (Fig. 1) the differences in H3K9/14diac accumulation were less prominent in the promoter regions encompassing the transcriptional start site (TSS) compared to downstream transcribed regions. At least for the *HY5* locus, HY5 occupancy in the promoter region increases upon UV-B illumination (Binkert et al. 2014) whereas UVR8 has been detected on both the promoter and transcribed region (Cloix and Jenkins 2008). If HY5 is the direct physical recruiter of the acetylation machinery, it might be expected that the modification would occur at some distance from the binding site of the transcription factor due to steric constraints, consistent with the observed pattern. On the other hand, the presence of UVR8 on the chromatin of these distant sites could contribute to the structural integrity of the histone modifying complex by providing interaction sites for its peripheral subunits. It is currently unclear why only some target loci and not others have been observed to display the UVR8-chromatin interaction in ChIP experiments (Cloix and Jenkins 2008), but as is the case with many assays, ChIPs have certain detection limits that may leave unnoticed some weak/transient, yet true and physiologically effective chromatin associations. Thus, it remains unclear whether the observed association of UVR8 with chromatin has functional significance.

Recent studies have highlighted the importance of elucidating the functional significance of crosstalk among co-existing histone marks (Schwammle et al. 2014). Since UVR8 is known to associate preferentially with histone H2B in vitro, one intriguing option was to check whether monoubiquitination of H2B is an important histone modification in UV-B responses. H2Bub has been shown to be related to actively transcribed genes during photomorphogenesis (Bourbousse et al. 2012) and has also been reported to have high association with H3K4me3 along the Arabidopsis genome (Roudier et al. 2011). In addition, H3K4me3 has been shown to significantly correlate with H3K9ac on the same loci, suggesting a mechanism of controlling gene expression changes via coordinated deposition of distinct histone marks (Ha et al. 2011; Jang et al. 2011). Furthermore, an additional candidate that appeared worthy of incorporation in our study was H3K36me3. This particular modification has been reported to be highly associated with H2Bub and H3K4me3 along the genome of Arabidopsis (Roudier et al. 2011). In yeast, it is essential for proper transcriptional elongation (Carrozza et al. 2005) and, interestingly, cycling of H2B between the monoubiquitinated and deubiquitinated states is essential for the sequential recruitment of the methyltransferases that mediate the deposition of the H3K4me3 and H3K36me3 histone marks (Weake and Workman 2008). In plants, the proposed model suggests that H3K4me3 appears prior to H2B ubiquitination and deubiquitination, whereas H3K36me3 occurs afterwards (Schmitz et al. 2009). None of these histone modifications were found to undergo noteworthy fluctuations of their abundance over selected UVR8-regulated loci following UV-B illumination (Supplementary Figs. S4–S7). Similar were the findings for H3K9me3, to which initial studies on the epigenetic regulation of vernalization had ascribed a repressive function along with H3K27me3 (Schmitz et al. 2009). Subsequent research, however, placed H3K9me3 among the activating marks (Charron et al. 2009) that are predominantly involved in transcriptional elongation (Roudier et al. 2011). H3K56ac is commonly found on short domains residing around the 5′ end of the transcribed regions of expressed genes (Tanurdzic et al. 2008) and it has been shown to appear in tight association with H3K4me3 (Roudier et al. 2011), which was readily detected in our ChIP experiments, although without displaying any interesting UVR8-mediated fluctuations upon UV-B illumination. Moreover, studies in yeast have implicated H3K56ac with the recruitment of chromatin remodelling factors and gene activation (Xu et al. 2005). Our results revealed interesting enrichment patterns (Supplementary Fig. S8), and two biological repetitions were sufficient to highlight clear differences. However, more experiments would be necessary to resolve borderline effects.

The experiments with AA provide additional evidence for the involvement of histone acetylation in UVR8-mediated regulation of transcription. AA inhibited both UV-B induced transcription of the UVR8-regulated *ELIP1* and *CHS* genes and UV-B-induced H3K9,14diac enrichment at the corresponding genomic loci. The lesser effect of AA on the expression of *HY5* may be explained by differences in the regulation of UVR8-target genes. *HY5* is an early-expressed gene that is subject to autoregulation (Binkert et al. 2014) and is required for the UV-B induced transcription of genes such as *ELIP1* and *CHS* (Brown et al. 2005; Favory et al. 2009). Thus, transcription of the genes could involve different histone acetylation activities, which differ in susceptibility to AA inhibition. Moreover, as transcription is strongly influenced but not determined by histone acetylation, it should be kept in mind that additional factors, including other chromatin modifications, may influence the transcription of different genes.

In an attempt to gain a deeper understanding of the nature of UVR8 involvement in the epigenetic processes it regulates, we first investigated the possibility of direct physical interactions with specific HATs and HDACs (Supplementary Figs. S10–S13). There are at least 10 different HAT-A (acetylate nucleosomal core histones) acetyltransferases in Arabidopsis, and they are grouped in four (Pandey et al. 2002) or five (Boycheva et al. 2014) families. Arabidopsis appears to have no <17 different individual HDACs (Ma et al. 2013). Essentially, therefore, our strategy had to rely on selective, targeted attempts, against specific members that appeared relevant to our working hypothesis in the light of published information (Ausín et al. 2004; Barneche et al. 2014; Benhamed et al. 2006, 2008; Bertrand et al. 2005; Campi et al. 2012; Fisher and Franklin 2011; Pazhouhandeh et al. 2011). Nevertheless, with the possible exception of FVE, for which the weak interaction with UVR8 (Supplementary Fig. S12) needs further validation via an *in planta* approach, no direct physical interactions were detected in yeast. Similarly, no interactions were observed with HY5. These negative results might be a consequence of the fact that histone acetylation and/or deacetylation are commonly performed by large protein complexes, where the subunits with the actual catalytic activity are surrounded by numerous adaptor proteins which contribute to the structural stability of the complex. However the *in planta* assays we performed did not reveal any evidence of the involvement of the corresponding proteins in UVR8-mediated responses (Fig. 6). Of course, this conclusion only applies to the particular tested responses, and it is possible that, had other UV-B-related phenotypic traits been investigated, clearly observable differences might have been seen. For example, the inhibition of primary root elongation by UV-B (Tong et al. 2008) has been used to demonstrate that *fve* plants are less responsive to UV-B than the wild-type (Campi et al. 2012). Moreover, potential functional redundancy and/or functional diversification among related histone modifying enzymes could be a crucial factor affecting the outcome of the in vivo experiments. Recently it was reported that HAG3 participates in UV-B-induced DNA damage repair and signalling, whereas the other two GNAT family HATs, namely HAG1 (GCN5) and HAG2, have less important roles but might still be involved in some aspects of UV-B signalling (Fina and Casati 2015). Furthermore, our studies have only examined a limited number of the potential chromatin modifiers and associated proteins that could conceivably act with UVR8 in UV-B responses. Ultimately, for direct links between the HATs/HDACs of interest and the deposition of the UV-B-induced accumulation of H3K9,14diac over specific UVR8-regulated genetic loci to be established, ChIP assays with the corresponding mutants would be needed. Global alterations in the histone acetylation levels in such mutant lines would need to be taken into consideration when interpreting the results, but clearly observable differences could in principle be detected.

In conclusion, the data presented in this work are consistent with previous reports of a role for histone acetylation in photomorphogenic responses. The mechanism of transcriptional regulation is the least understood facet of photomorphogenic UV-B signalling, and the data presented show that UVR8-dependent epigenetic processes are involved, specifically with the enrichment of H3K9,14diac at UVR8-regulated genomic loci. The ChIPseq dataset can serve as a valuable resource allowing researchers to correlate UV-B-induced transcriptional responses with histone acetylation profiles. While our results do not establish the mechanistic details of UVR8 involvement, they do provide a good reference point for future research. We propose a model (Fig. 7) in which both HY5 and UVR8 are involved in the recruitment of the histone modifying machinery. Although our findings indicate that p300/CBP-PCAF acetylation activities are involved (Fig. 5), the identity of the catalytic subunits as well as of the peripheral adaptors remains to be discovered. Our findings do not provide evidence for the direct, physical involvement of UVR8 or HY5 in recruiting histone modifiers to a chromatin-associated complex. But neither do they refute such a model. Further research is therefore required to determine whether or not the association of UVR8 with chromatin, however weak and elusive with available detection sensitivities, is functionally significant.

## Electronic supplementary material

Below is the link to the electronic supplementary material.

Supplementary material 1 (PDF 4080 KB)
