# Supplementary material for: Regulation of transcription by the Arabidopsis UVR8 photoreceptor involves a specific histone modification
Source: Plant Mol Biol. 2016 Aug 17;92(4):425–43. doi: 10.1007/s11103-016-0522-3 (PMC5080334; doi:10.1007/s11103-016-0522-3)
Supplement: Supplementary file 1 — Supplementary material 1 (PDF 4080 KB) [file 11103_2016_522_MOESM1_ESM.pdf]

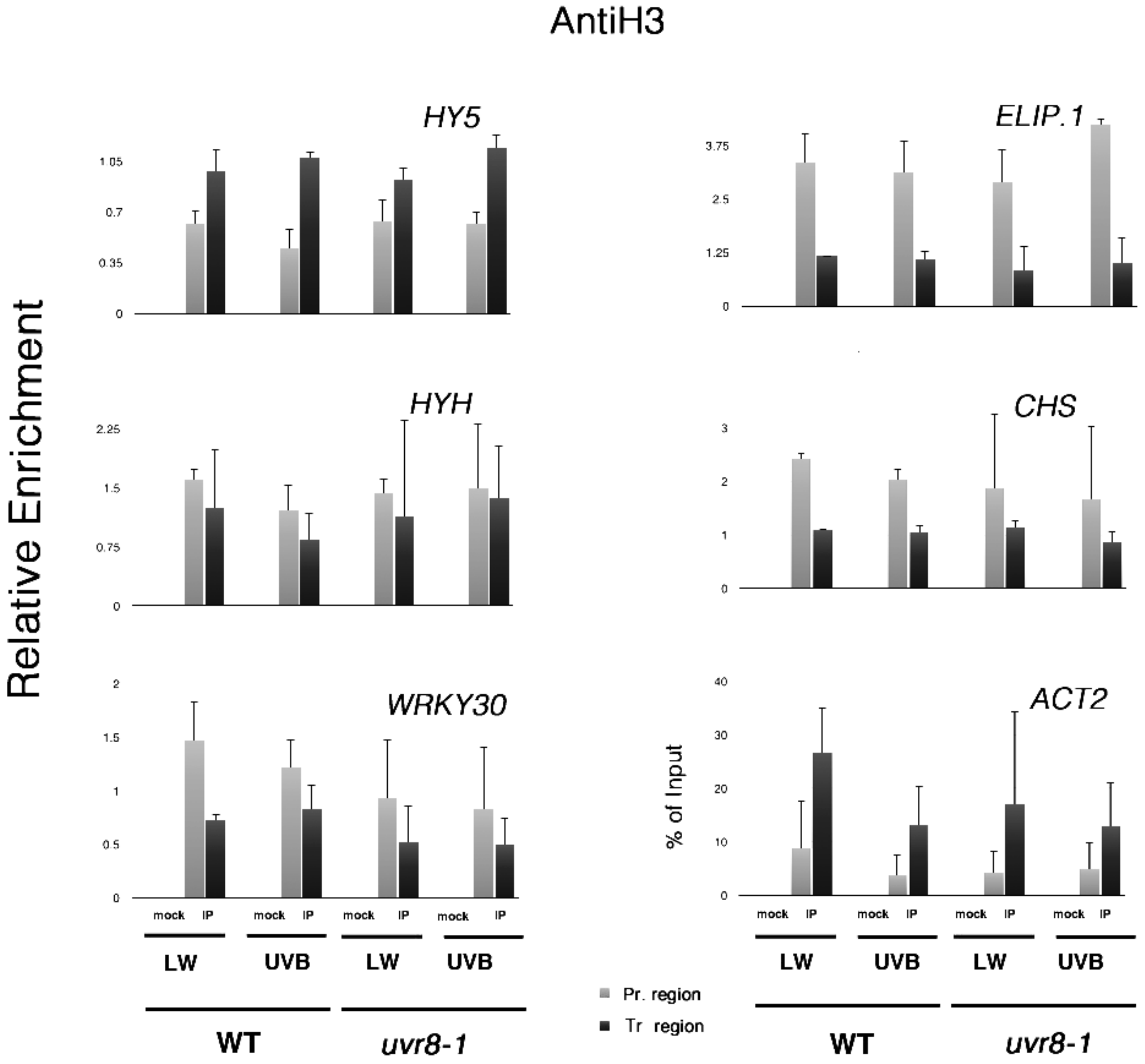

Figure S1. UV-B does not cause ChIP - detectable alterations in nucleosome density in the assayed genetic loci.

Each graph displays the relative enrichment in histone H3 over promoter (gray) and transcribed region (black) DNA of the designated genes, expressed as % of Input normalised against *ACT2*. For *ACT2* itself, no normalisation was performed and enrichment is given as % of Input. Immunoprecipitation was performed with antibody against an invariant domain of histone H3. Wild-type (WT) and *uvr8-1* plants were exposed to UV-B or control light treatments. LW: low fluence rate white light (15 μmol $m^{-2} s^{-1}$). UVB: 1.5 μmol $m^{-2} s^{-1}$ narrow band UV-B for 4 h. Mock: No Ab control. IP: Immunoprecipitated material. Error bars represent SD from two independent experiments.

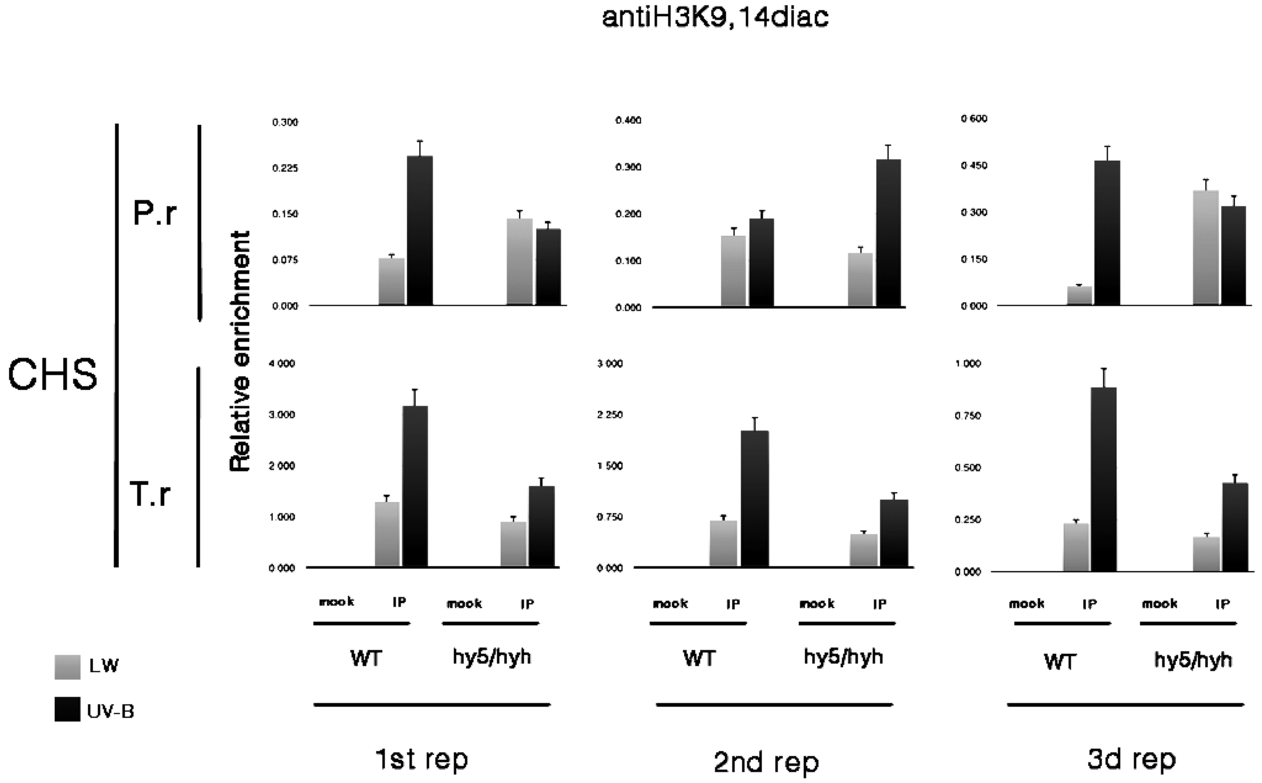

Figure S2. UV-B caused enhancement of H3K9,14diac levels over a transcribed region of *CHS,* and HY5 and/or *HYH* seem to be required for a full magnitude response.

Each graph displays the relative enrichment over promoter or transcribed region of CHS. ChIPed DNA is expressed as % of Input normalised against *ACT2*. Wild-type and *hy5/hyh* plants were exposed to UV-B or control light treatments. LW: low fluence rate white light (15 µmol m^{-2} s^{-1}). UV-B: 1.5 µmol m^{-2} s^{-1} narrow band UV-B for 4 h. Mock: No Ab control. IP: Immunoprecipitated material. Three independent biological repetitions are presented individually and error bars on each graph indicate SE from three technical replicates.

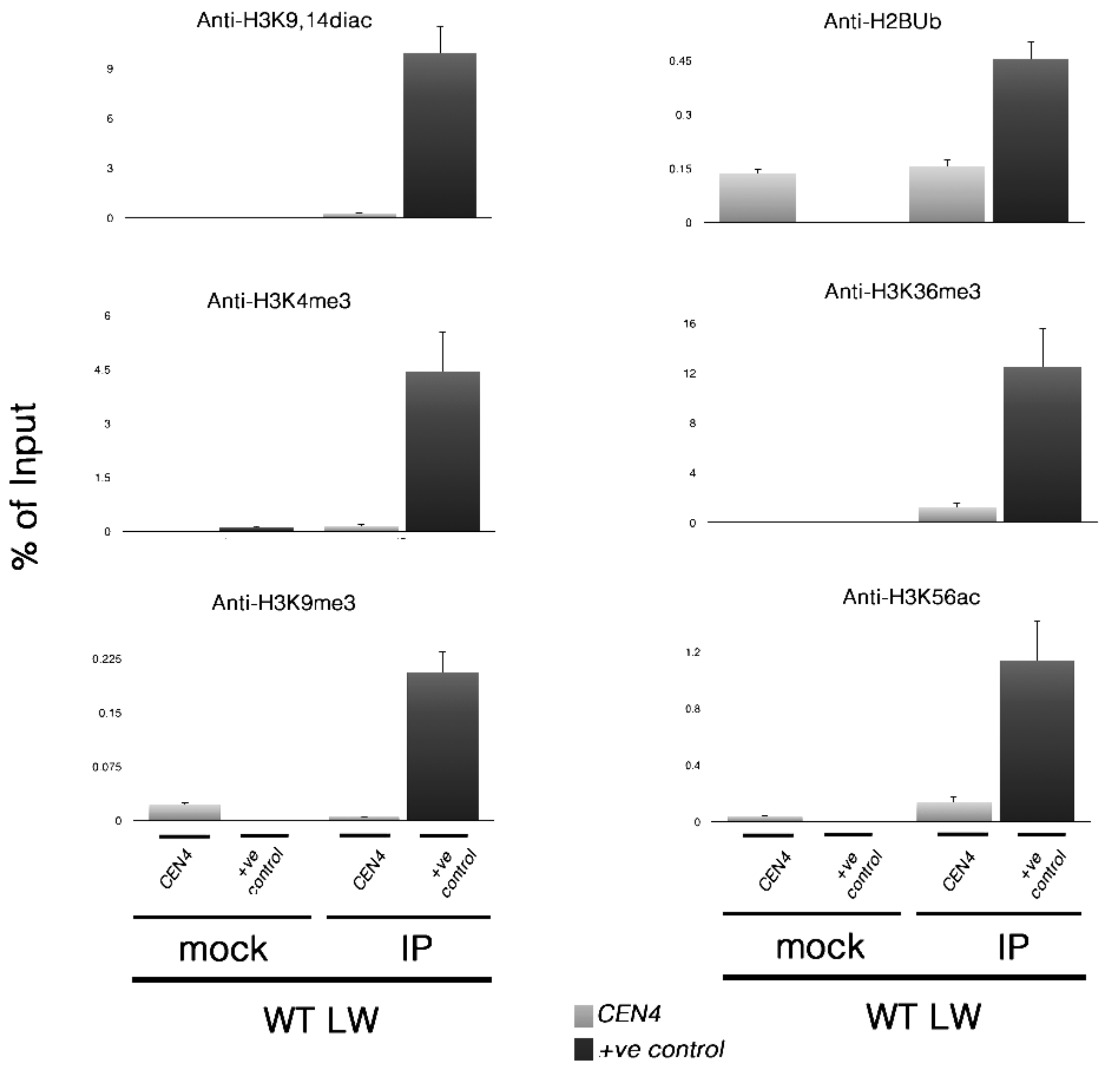

Figure S3. Non-target chromatin is not present in the immunoprecipitates.

Each graph displays the enrichment for the designated histone marks, presented as % of Input, over two sequences; a heterochromatin-associated centromeric sequence, *CEN4* (gray) and a +ve control sequence (black). The +ve control sequence was *UBQ5* for H3K9me3 and *ACT2* for the rest. Experiments were performed only for wild-type (WT) plants under low fluence rate white light (LW) 15 µmol m^{-2} s^{-1}. Mock: No Ab control. IP: Immunoprecipitated material. Error bars represent SD from two independent experiments.

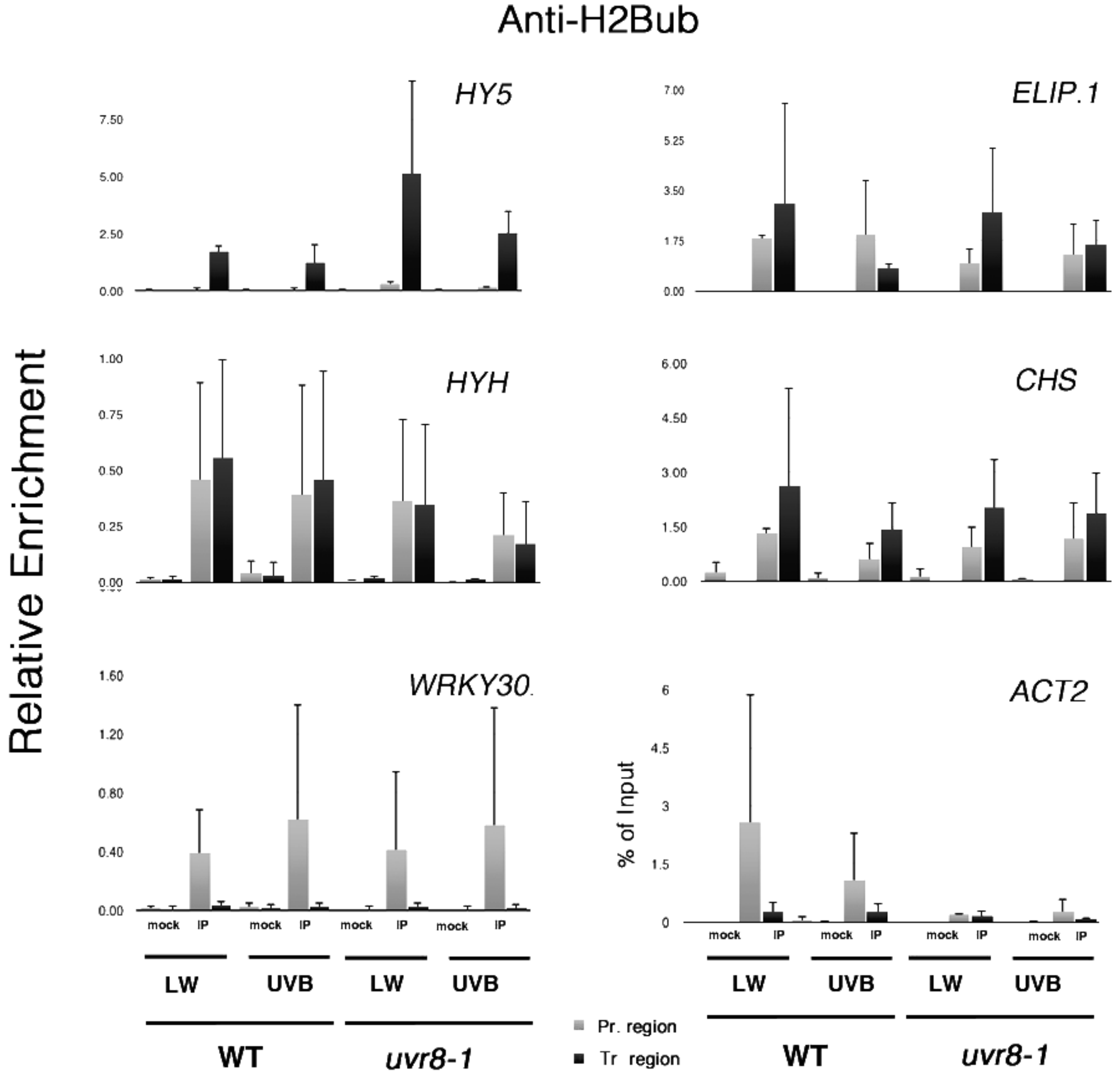

Figure S4. Results of ChIP with Anti-H2Bub antibody.

Each graph displays the relative enrichment in H2Bub, for both wild-type (WT) and *uvr8-1*, on promoter (gray) and transcribed regions (black) of the designated genes. LW: low fluence rate white light (15 µmol m^{-2} s^{-1}). UVB: 1.5 µmol m^{-2} s^{-1} narrow band UV-B for 4 h. Mock: No Ab control. IP: Immunoprecipitated material. Results are expressed as % of Input normalised against *ACT2*. For *ACT2* itself, no normalisation was performed and enrichment is given as % of Input. Error bars represent SD from two independent biological replicates.

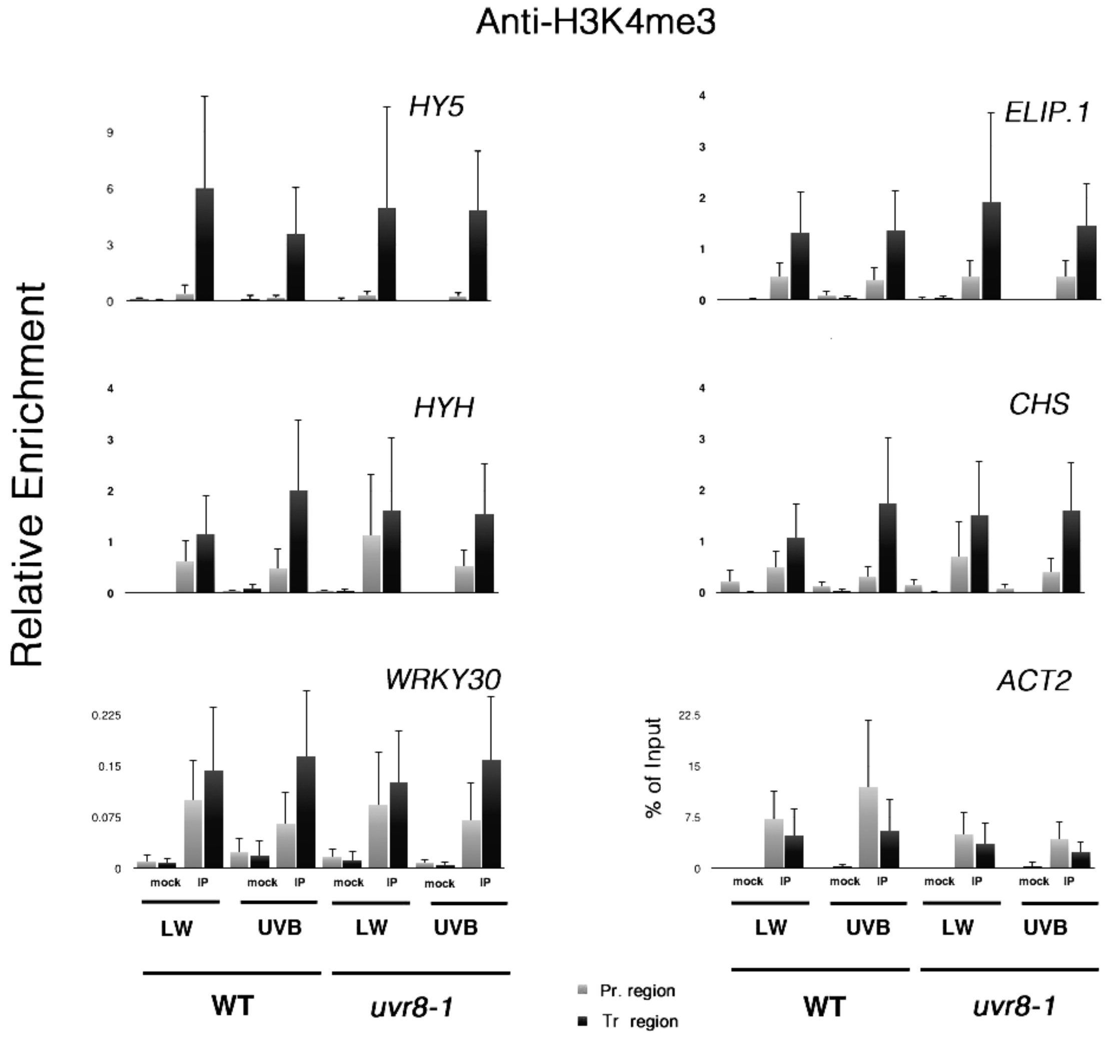

Figure S5. Results of ChIP with Anti-H3K4me3 antibody.

Each graph displays the enrichment in trimethylated Lys 4 of H3 for both wild-type (WT) and *uvr8-1* plants, under low white light and UV-B irradiation, for promoter (gray) and transcribed region (black) of the designated genes. LW: low fluence rate white light (15 µmol m^{-2} s^{-1}). UVB: 1.5 µmol m^{-2} s^{-1} UV-B for 4 h. Mock: No Ab control. IP: Immunoprecipitated material. Results are expressed as % of Input normalised against *ACT2*. For *ACT2* itself, no normalisation was performed and enrichment is given as % of Input. Error bars represent SD (n=3).

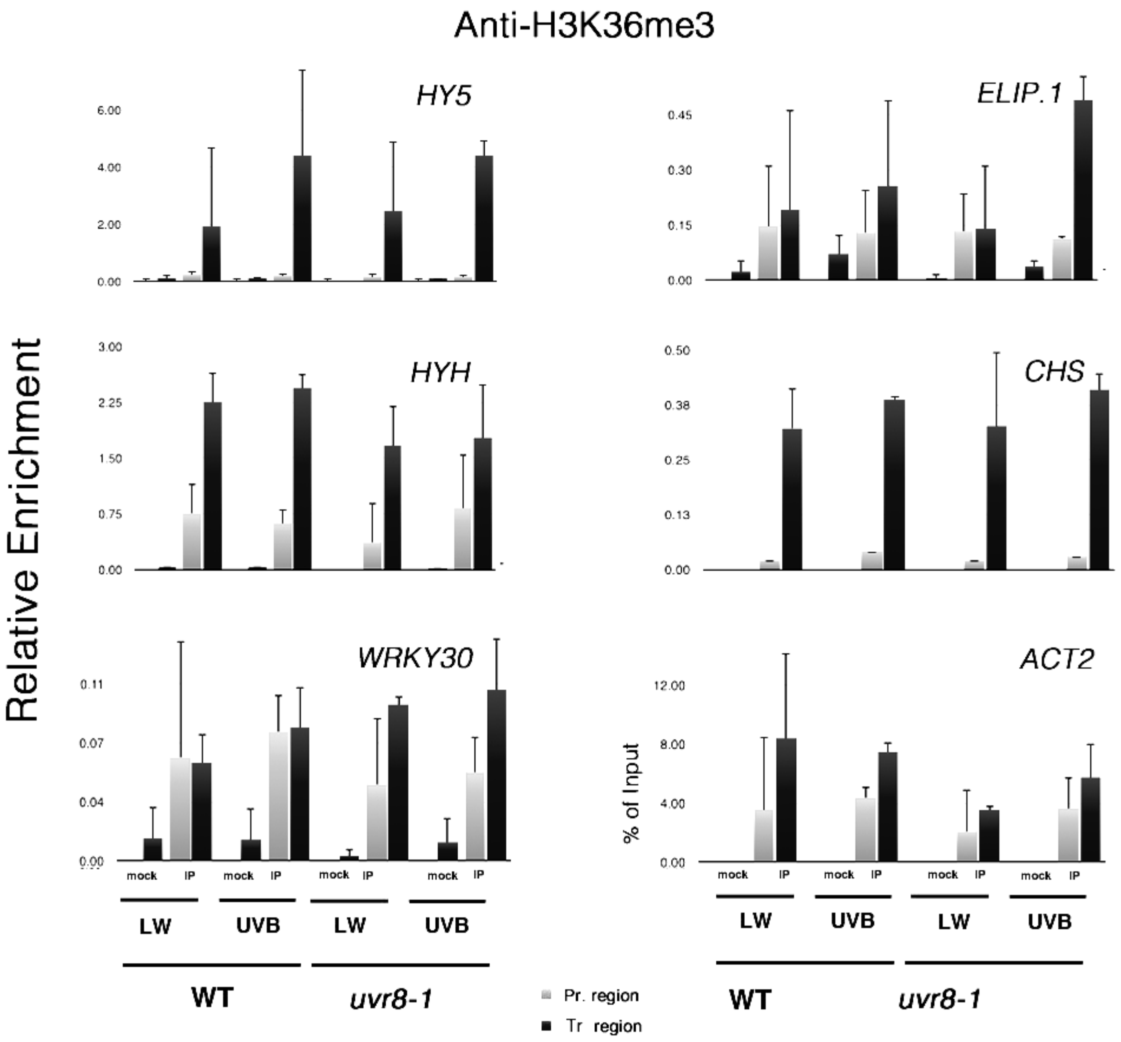

Figure S6. Results of ChIP with Anti-H3K36me3 antibody.

Each graph displays the enrichment in trimethylated Lys 36 of H3 for both wild-type (WT) and *uvr8-1* plants, under low white light and UV-B irradiation, for promoter (gray) and transcribed region (black) of the designated genes. LW: low fluence rate white light (15 µmol m^{-2} s^{-1}). UVB: 1.5 µmol m^{-2} s^{-1} UV-B for 4 h. Mock: No Ab control. IP: Immunoprecipitated material. Results are expressed as % of Input for *ACT2*, and normalised % of Input against *ACT2* for all other genes. Error bars represent SD from two independent biological replicates.

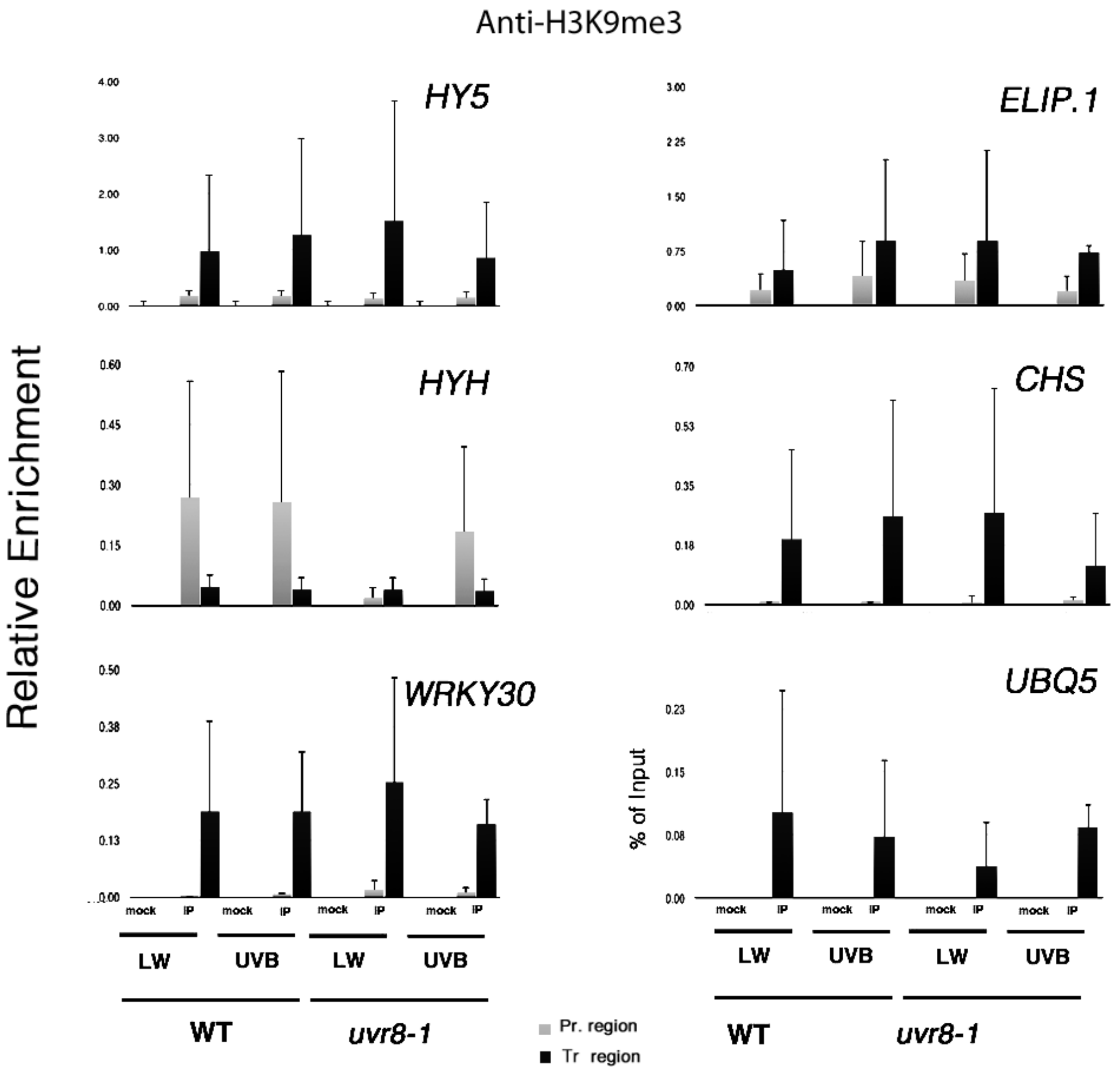

Figure S7. Results of ChIP with Anti-H3K9me3 antibody.

Each graph displays the enrichment in H3K9me3 for both wild-type (WT) and *uvr8-1* plants, under low white light and UV-B irradiation, for promoter (gray) and transcribed region (black) of the designated genes. LW: low fluence rate white light (15 μmol m^{-2} s^{-1}). UVB: 1.5 μmol m^{-2} s^{-1} UV-B for 4 hours. Mock: No Ab control. IP: Immunoprecipitated material. Results are expressed as % of Input normalised against *UBQ5*. For *UBQ5* itself, no normalisation was performed and enrichment is given as % of Input. Error bars represent SD from two independent biological replicates.

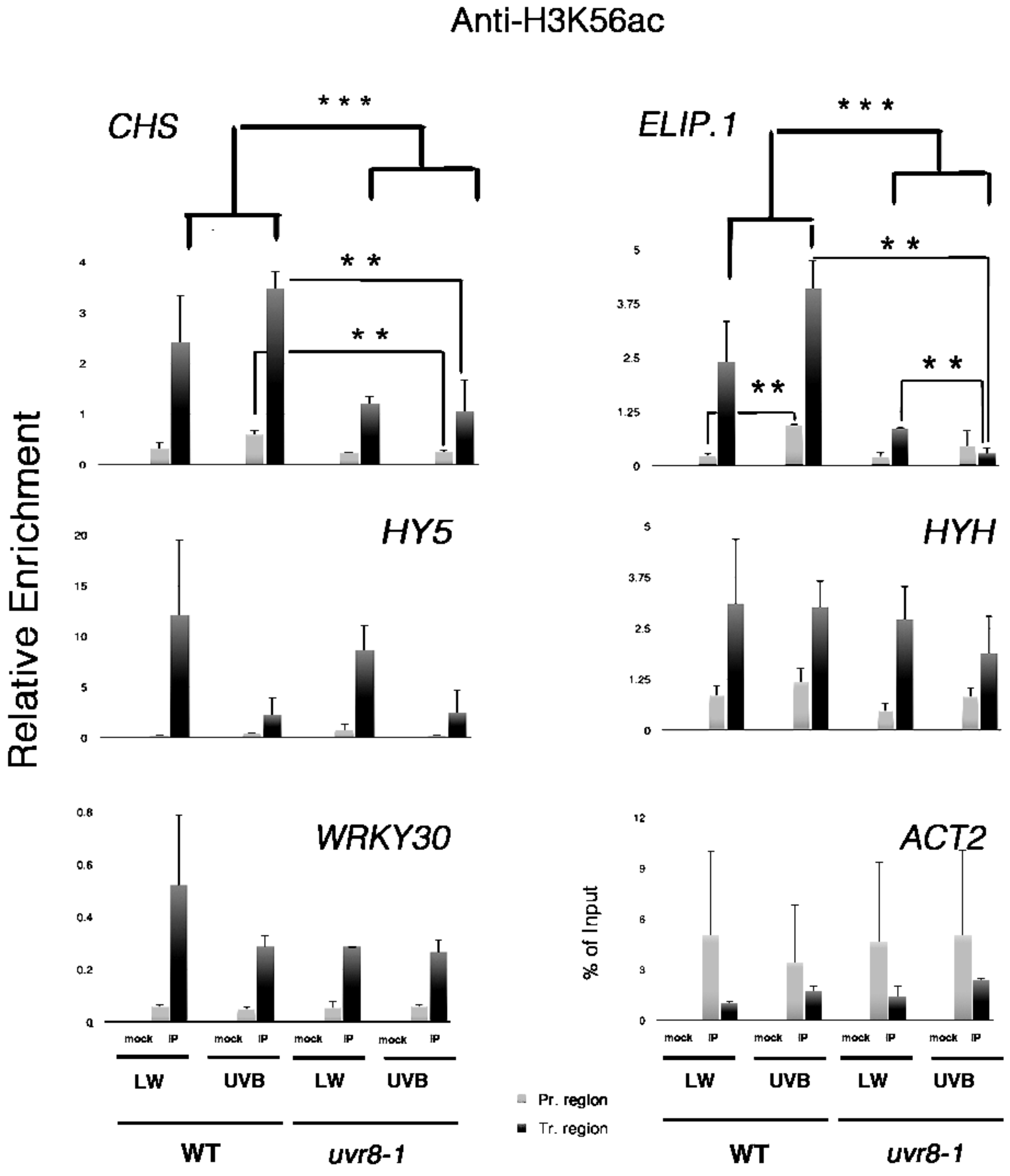

Figure S8. UVR8 might be linked to a locus-specific accumulation of H3K56ac, for a subset of the UVR8-dependent UV-B responsive genes.

Each graph displays the enrichment in H3K56ac for both wild-type (WT) and *uvr8-1* plants, under low fluence rate white light and UV-B irradiation, for promoter (gray) and transcribed region (black) of the designated genes. LW: low fluence rate white light (15 µmol m^{-2} s^{-1}). UVB: 1.5 µmol m^{-2} s^{-1} UV-B for 4 h. Mock: No Ab control. IP: Immunoprecipitated material. Results are expressed as % of Input normalised against *ACT2*. For *ACT2* itself, no normalisation was performed and enrichment is given as % of Input. Error bars represent SD from two independent biological replicates. ** : $p \leq 0.05$, *** : $p \leq 0.01$

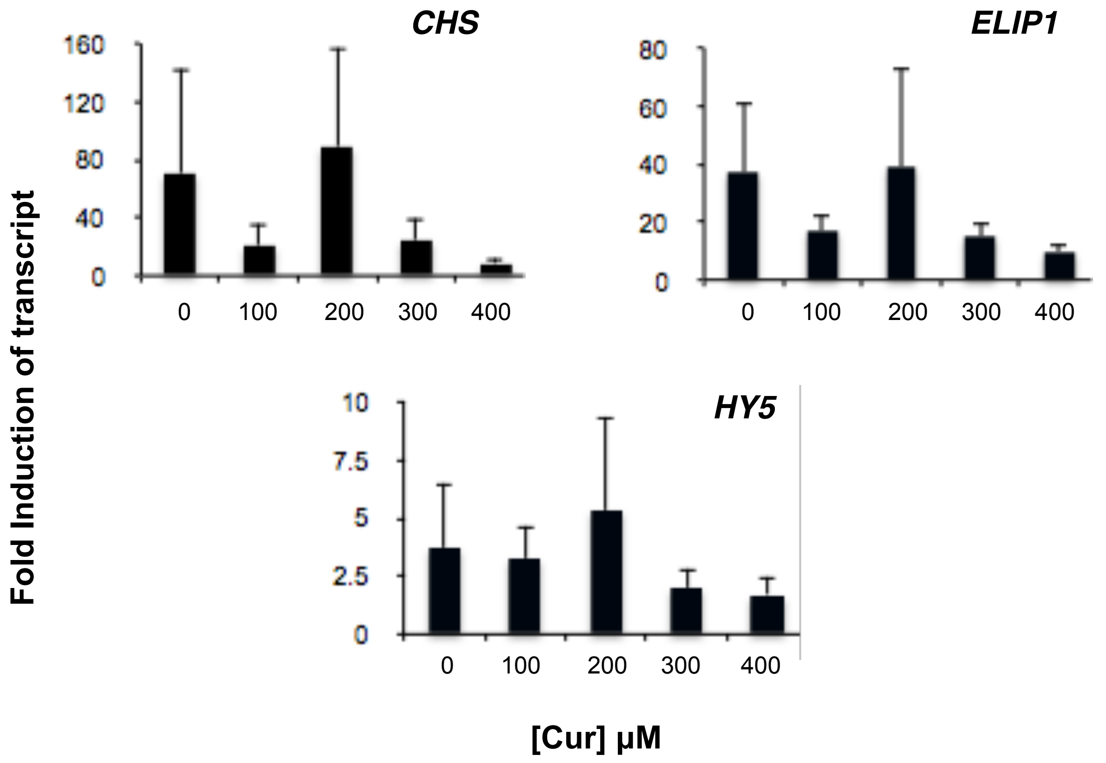

Figure S9. Effect of curcumin on expression of specific UVR8-regulated genes.

Fold UV-B induction of transcripts of *CHS*, *ELIP1* and *HY5*, measured by RT-qPCR, in plants treated with increasing concentrations of curcumin (Cur). Plants were infiltrated with the inhibitor for 15 mins and then exposed (or not in controls) to 1.5 µmol m^{-2} s^{-1} narrowband UV-B for 1 hour. Plants were harvested after 2 hours in darkness for RT-qPCR. Error bars represent SD (n=3).

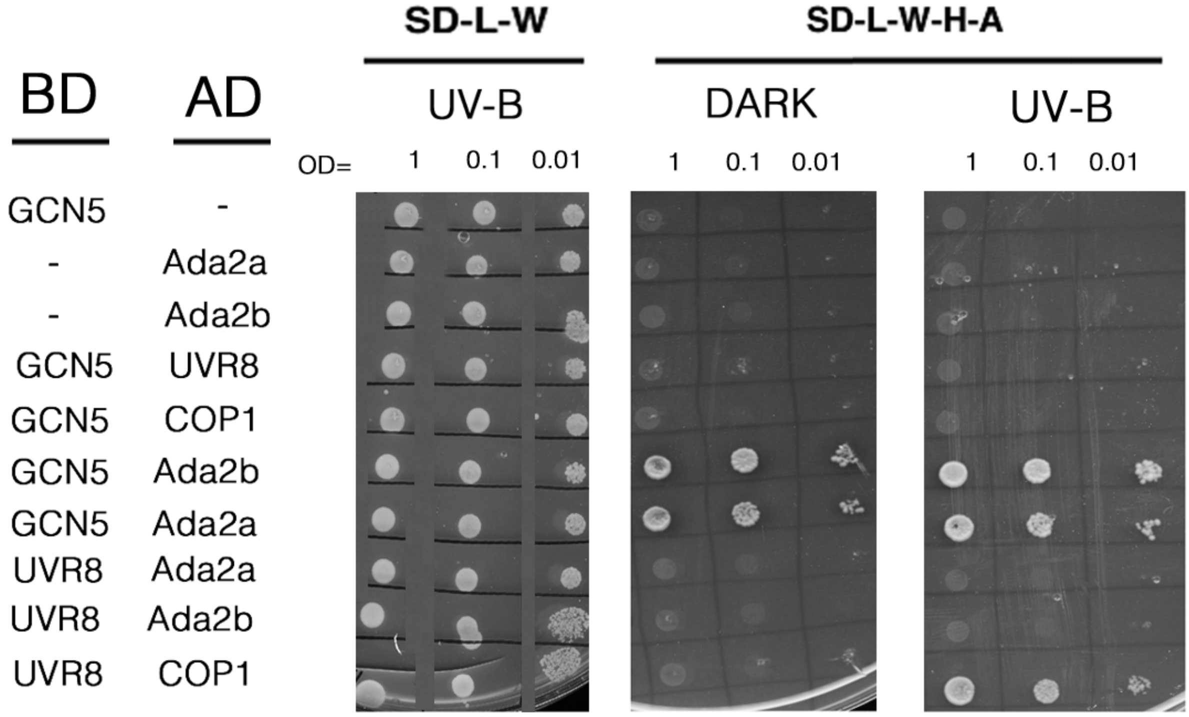

Figure S10. Neither GCN5 nor its interacting partners ADA2a and ADA2b interact with UVR8 in yeast.

The UVR8-COP1 interaction was used as a control of the effectiveness of the UV-B treatment, whereas the interactions between GCN5 and the two ADA2 proteins were employed to show that all constructs behave as expected. SD-L-W medium was used as a control for the viability of the spotted cells. Interactions were assessed only on high stringency selection plates (SD-L-W-H-A). Yeast spottings were performed from cell suspensions with three different cell densities (OD=1, 0.1 and 0.01). Results are representative of three independent biological replicates.

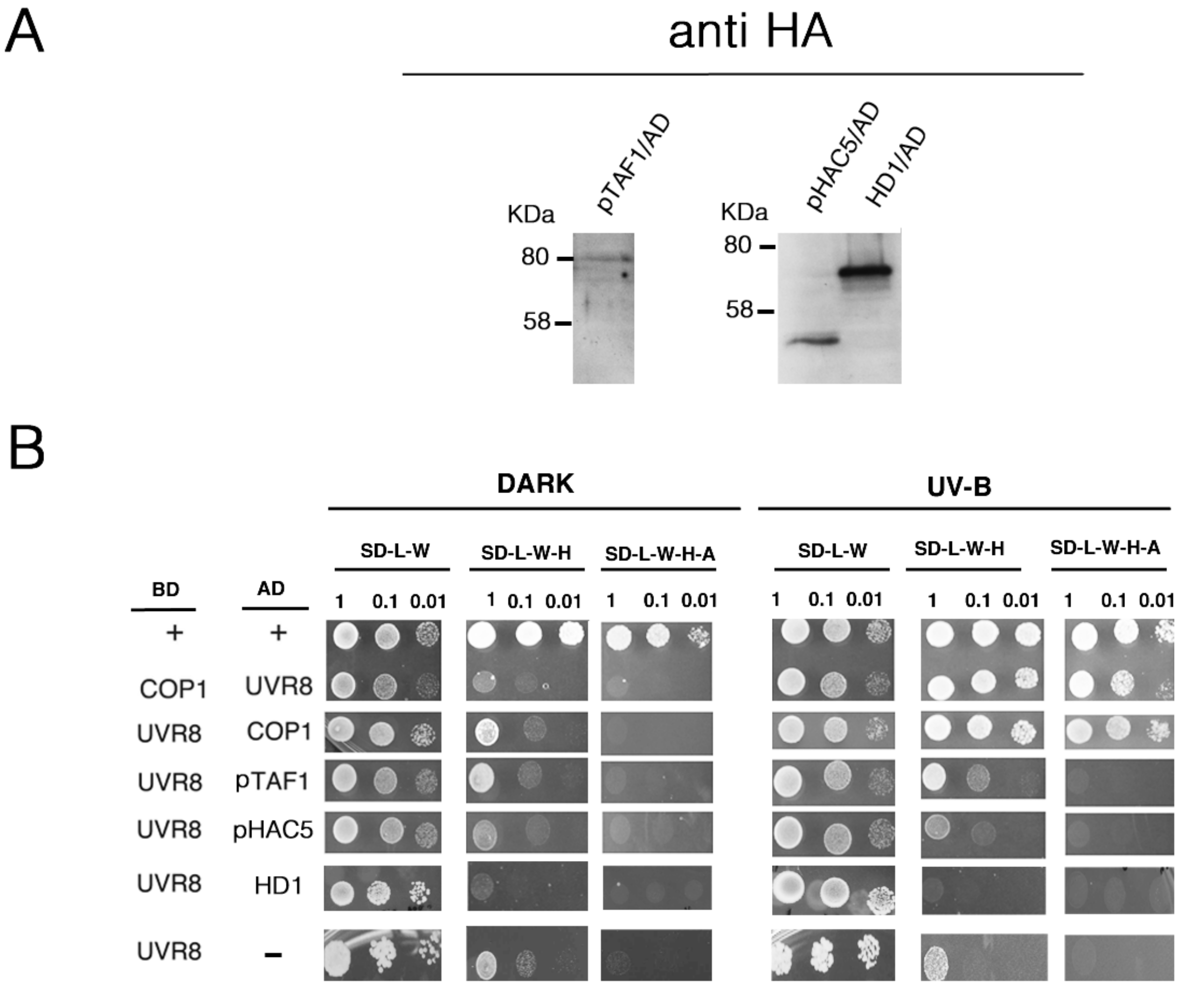

Figure S11. UVR8 does not interact with HD1, a partial sequence of TAF1 (pTAF1) or the C-terminal sequence of HAC5 (pHAC5).

(a) Immunoblots with anti-HA antibody for detection of the recombinant proteins of interest in the yeast cells. (b) The interaction between the murine p53/GAL4BD fusion protein (+BD) and the SV40 large T-antigen/GAL4AD fusion protein (+AD) was used as a general positive control for the Y2H assay. The interaction between UVR8-COP1 interaction was used as a control for the effectiveness of the UV-B treatment. Because of the large size of the full length *TAF1* and *HAC5* cDNAs, partial sequences encoding domains known to mediate protein-protein interactions were used for the assay. SD-L-W medium was used as a control for the viability of the spotted cells. Interactions were assessed on low (SD-L-W-H) and high (SD-L-W-H-A) stringency selection plates. Yeast spottings were performed from cell suspensions with three different cell densities (OD=1, 0.1 and 0.01). Results are representative of three repeats.

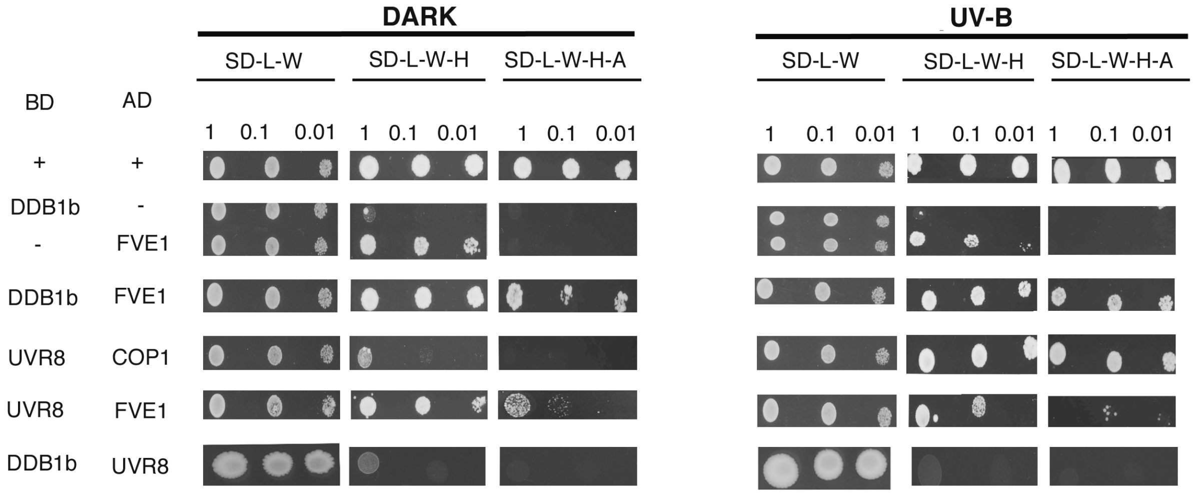

Figure S12. UVR8 appears to interact weakly with FVE.

The interaction between the murine p53/GAL4BD fusion protein (+BD) and the SV40 large T-antigen/GAL4AD fusion protein (+AD) was used as a general positive control for the Y2H assay. The interaction between UVR8-COP1 interaction was used as a control for the effectiveness of the UV-B treatment. The control interaction between FVE and DDB1b (Pazhouhandeh et al. 2011) was employed to demonstrate proper behaviour of the recombinant proteins in yeast. SD-L-W medium was used as a control for the viability of the spotted cells. Interactions were assessed on low (SD-L-W-H) and high (SD-L-W-H-A) stringency selection plates. Yeast spottings were performed from cell suspensions with three different cell densities (OD=1, 0.1 and 0.01). Results are representative of two repeats. It should be noted, however, that that the assays were repeated four times and in two occasions the yeast growth on full selective medium was too slow to be convincingly regarded as indicative of a UVR8-FVE association. Such variations are commonly observed in Y2H assays for borderline interactions, and are usually attributed to differences in the expression levels of the proteins within the yeast cells and/or to batch-to-batch differences in the quality and concentrations of the supplemented dropout mediums. The UVR8-FVE interaction, therefore, needs further confirmation with alternative methodologies.

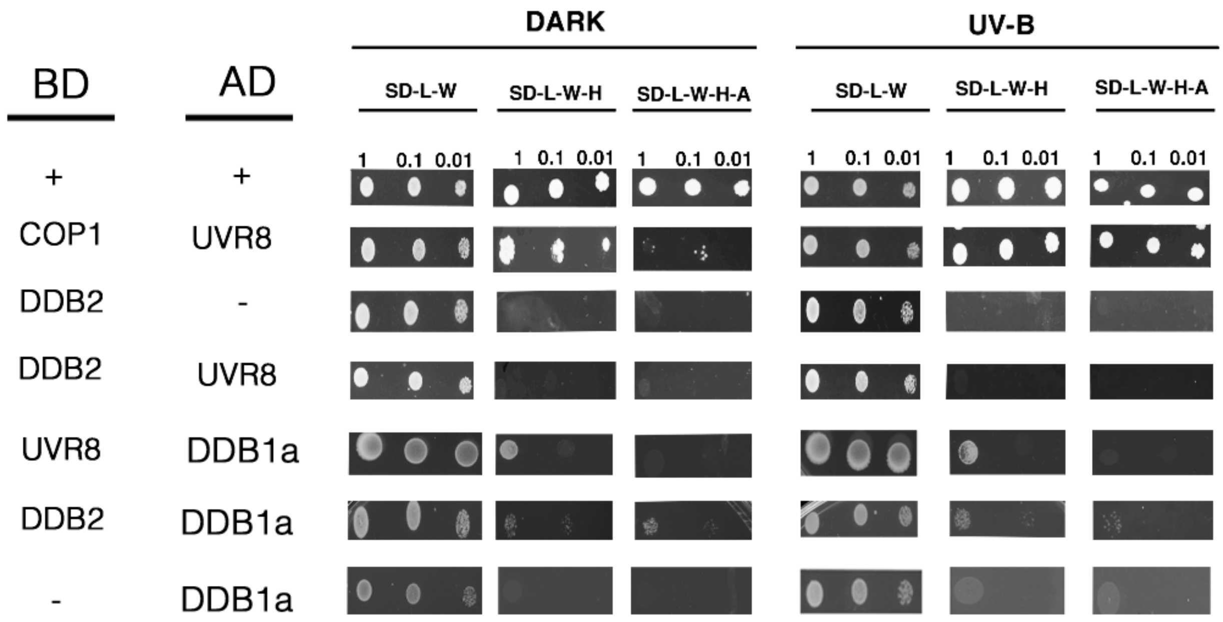

Figure S13. UVR8 does not interact with DDB2 or DDB1a in yeast.

The interaction between the murine p53/GAL4BD fusion protein (+BD) and the SV40 large T-antigen/GAL4AD fusion protein (+ AD) was used as a general positive control for the Y2H assay. The COP1-UVR8 interaction was used as a control for the effectiveness of the UV-B treatment. The anticipated weak interaction of DDB2 with DDB1a in yeast (Dumbliauskas et al. 2011) was employed to demonstrate that the recombinant proteins behaved as expected. SD-L-W medium was used as a control for the viability of the spotted cells. Interactions were assessed on low (SD-L-W-H) and high (SD-L-W-H-A) stringency selection plates. Yeast spottings were performed from cell suspensions with three different cell densities (OD=1, 0.1 and 0.01). Results are representative of three independent repeats.

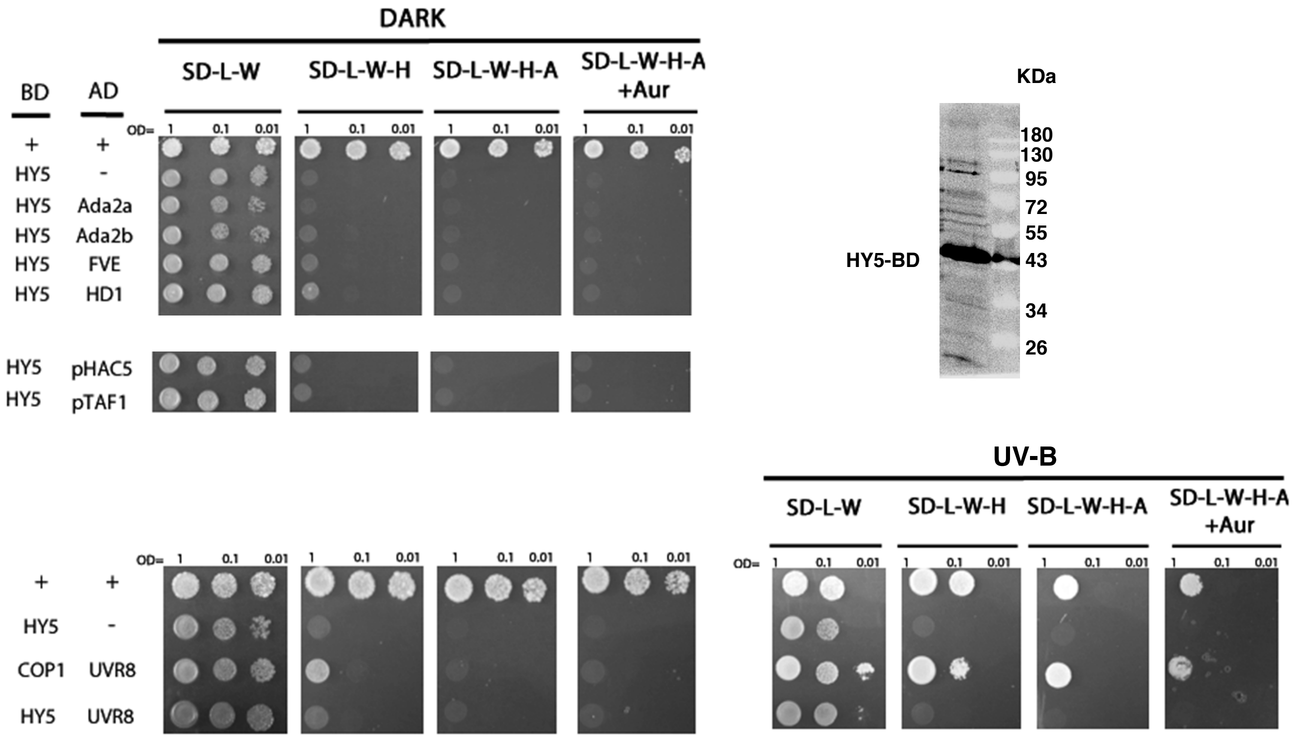

Figure S14. HY5 does not interact with UVR8 or major HATc/HDACs of interest.

The yeast strain used was Y2HGold (Clontech). The interaction between the murine p53/GAL4BD fusion protein (+BD) and the SV40 large T-antigen/GAL4AD fusion protein (+AD) was used as a general positive control for the Y2H assay. The interaction between UVR8 and COP1 was used as a control for the effectiveness of the UV-B treatment. SD-L-W medium was used as a control for the viability of the spotted cells. Interactions were assessed on low (SD-L-W-H), high (SD-L-W-H-A), and very high stringency selection plates (SD-L-W-H-A+Aur [Aureobasidin]) according to instructions from the MatchmakerTM Gold Yeast Two-Hybrid System User Manual (Clontech). Yeast spottings were performed from cell suspensions with three different cell densities (OD=1, 0.1 and 0.01). Immunoblot panel shows that HY5-BD construct could readily be detected in yeast protein extracts.

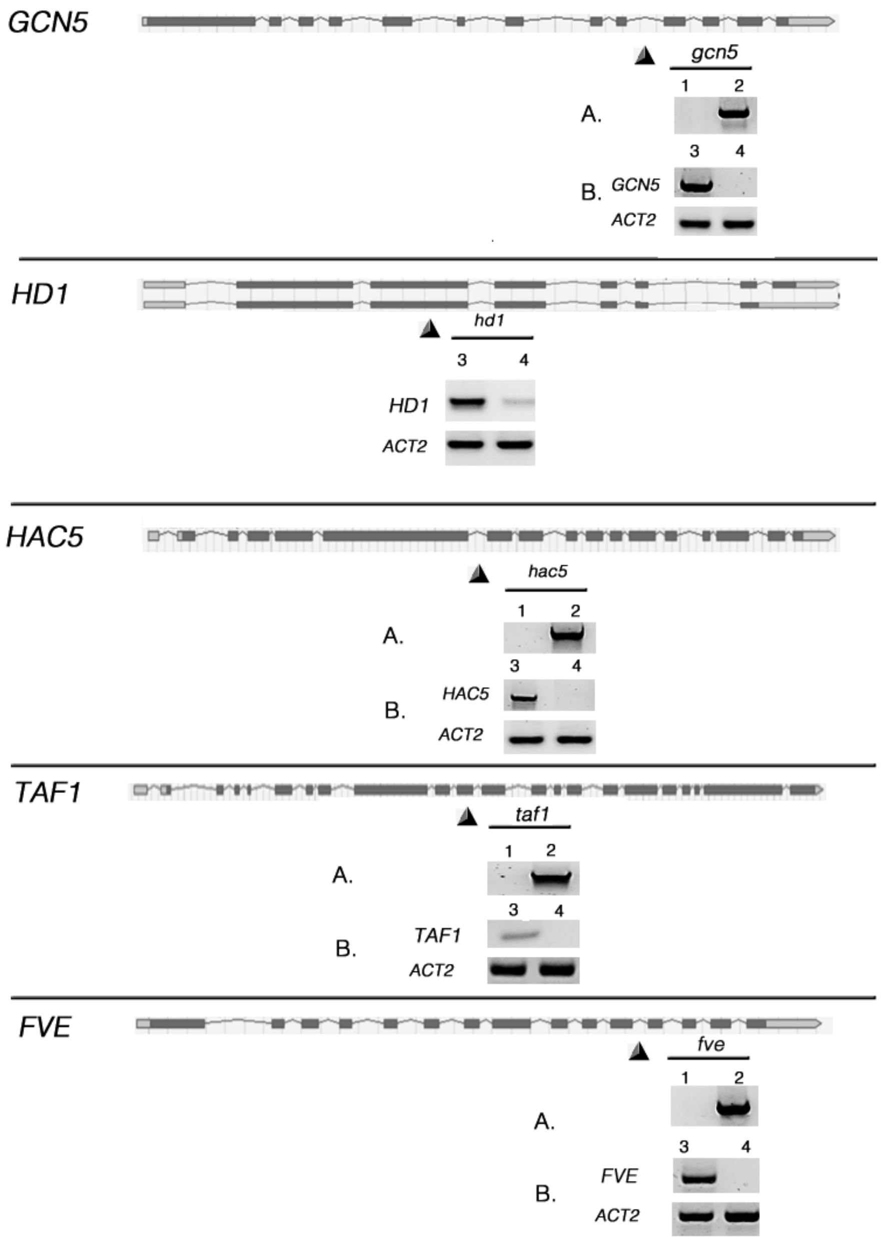

Figure S15. Identification of T-DNA insertion mutant lines for the HATs and HDACs of interest.

A genome browser view of each gene is presented, and the approximate location of every T-DNA insertion (according to TAIR database) is annotated with a triangle. Exons are depicted as dark grey thick lines, untranslated regions as light gray thick lines and promoters or introns as slim lines. For each gene, Panel A shows the genotyping results. Lane 1 corresponds to the PCR outcome when primers flanking the T-DNA insertion were used, and it should not give product if an insertion is present; Lane 2 shows the PCR product when a Left Border T-DNA-specific primer and a locus-specific primer are used. Panel B shows the results of RT-PCR using gene specific primers, which amplify the full length coding sequence of each gene, and *ACT2* primers as a control. Lane 3 : WT cDNA Lane 4 : mutant line cDNA.

Antibody	Application & Working Dilution	Source (Company and Cat Number, or Principal Ivestigator)
anti-H3	ChIP, 1/200	Abcam, Cat # : ab1791
anti-H3K9,14 Diac	ChIP and ChIPseq, 1/200	Millipore, Cat # : 06-599
anti-H3K4me3	ChIP, 1/200	Active Motif,<br>Cat # 39159 - 60
anti-H2Bub	ChIP, 1/200	Dr Ali Shilatifard<br>Northwestern University Feinberg School of Medicine. Chicago USA
anti-H3K36me3	ChIP, 1/200	Abcam, Cat # : ab9050
anti-H3K9me3	ChIP, 1/200	Millipore, Cat # : 07-442
anti-H3K56	ChIP, 1/200	Active Motif, Cat # : 39281
anti-CHS	WB 1/1000	Santa Cruz, Cat # : sc 12620
anti-HA	WB and Co-IP 1/5000	Abcam, Cat # : ab9110

Table S1 List of the antibodies used in this study

Primer sequences for ChIP	Primer Name
TCCCCTATCCATTATTCACCG	qChIP p.r *HY5* Fw
TTGCGAGACATTTTGGGAAGG	qChIP p.r *HY5* Rev
AGTTCAGGAAACAACTCGACC	qChIP p.r *ELIP.1*Fw
ATGTTGAACGATGCTGTTGCC	qChIP p.r ELIP.*1* Rev
CGAAATGATTCGTGTCTGTCG	qChIP p.r *ACT2* Fw
TGTTCTTCTCTGTCAAGTCGC	qChIP p.r *ACT2* Rev
AAGGATCGAGAAGCAGAGAAC	qChIP p.r *WRKY30* Fw
TTGCATGGCTTCTGGAAACTG	qChIP p.r *WRKY30* Rev
ACGAGTTGCAGACTTTGAGTG	qChIP p.r *HYH* Fw
CCAGTTTTGTGCTTCTTGTGG	qChIP p.r *HYH* Rev
CTAACCTACCACACTCTCATC	qChIP p.r *CHS* Fw
ATCCAAAGAAGAAGCACCAGC	qChIP p.r *CHS* Rev
TCCCAACGAGTGATCTCATTG	qChIP t.r *HY5* Fw
TTCTTTTCCGACAGCTTCTCC	qChIP t.r *HY5* Rev
AATGACCAGCTCGAAGAGAAG	qChIP t.r *HYH* Fw
CACTGAACAATGGATTAAAGGG	qChIP t.r *HYH* Rev
GTGAGCACAAAGTTTAGCGAC	qChIP t.r *ELIP.1* Fw
ACTTGGACTCAACGCTTATGC	qChIP t.r *ELIP.1* Rev
GTATTGTGCTGGATTCTGGTG	qChIP t.r *ACT2* Fw
GAGGTAATCAGTAAGGTCACG	qChIP t.r *ACT2* Rev
CAGACAGGACATCGTGGTGGT	qChIP t.r *CHS* Fw
ACATGAGTGATCTTTGACTTGG	qChIP t.r *CHS* Rev
TCGAAGAAGTCAATGCCAAGG	qChIP t.r *WRKY30* Fw
TCTCCAACTGAATCCATCGTC	qChIP t.r *WRKY30* Rev
ATAATCTTCAGCAGCCGTTGC	qChIP t.r *UBQ5* Fw
GAAAATCAATCGCTGCTGGTC	qChIP t.r *UBQ5* Rev
CACATCAGTCTGTCACCATCAAG	qCHIP *CEN4* Fw
CTACTCCAAATCTTACAAACCC	qCHIP *CEN* Rev
**Primer sequences for Y2H assay**	**Primer Name**
ATATTGAATTCATGGAAGAGATTTCGACGGA	*COP1*-pGBKT7 Fw
ATTGTCGACCTACTAGAATCACGCAGCGAGT	*COP1*-pGBKT7 Rev
AAAGAATTCATGAGCGTATGGAACTACGCC	*DDB1b*-pGBKT7 Fw
AAAGGATCCTCAGTGAAGCCTAGTGAGTTCTTCAAC	*DDB1b*-pGBKT7 Rev
AAACCATGGAAATGAGTTCAACGAGGAGCAG	*DDB2*-pGBKT7 Fw
TTTGTCGACTACATAACGACCTTCTTCACTC	*DDB2*-pGBKT7 Rev
AAAACATATGATGCAGGAACAAGCGACTAG	*HY5*-pGBKT7 Fw & *HY5*-pGADT7 Fw
AAAGTCGACAGAAGAAGAAGGAGATCAAAGG	*HY5*-pGBKT7 Rev
AAAAATCGATAGAAGAAGAAGGAGATCAAAGG	*HY5*-pGADT7 Rev
AATACATATGATGGAGAGCGACGAAGCAG	*FVE/MSI4* -pGADT7 Fw
TATAATCGATCTCTTAAGGCTTGGAGGC	*FVE/MSI4*-pGADT7 Rev
AAAGAATTCAGCTCATGGAACTACGTTGTTAC	*DDB1a*-pGADT7 Fw
ATATTATCGATGCGTTGATTGATGATTGATTTGAC	*DDB1a*-pGADT7 Rev
TTTCATATGCATTGTTGTACTCTCATGGTATCTG	partial*HAC5*-pGADT Fw
TTTATCGATTCATTCAGGAGTGGAGGCCGTTG	partial*HAC5*-pGADT7 Rev
ATTCCCGGGGGATACTGGCGGCAATTCGCTG	*HD1*-pGADT7 Fw
TATCTCGAGGAAATTAGAAGCTCCGAGTCTTATG	*HD1*-pGADT7 Rev
AGGCCATGGAGGCCATGAGTTCAACGAGGAGCAG	*DDB2*-pGADT7 FW
TTTCTCGAGTACATAACGACCTTCTTCACTC	*DDB2*-pGADT7 Rev
ATACATATGGGCTGTGGATCAAATTCTGGGTGG	partial*TAF1*-pGADT7-Fw
ATAATCGATTGCCTATCCCAAATCTCATGACAC	partial*TAF1*-pGADT7-Rev
**Primer sequences for Genotyping**	**Primer Name**
AAACGTCTTACCTGGTTGCAC	*gcn5* LP
ACGTATCAGTTTCTGATCCGG	*gcn5* RP
AGGCCAGGTAAGCTAACGAAG	*hac5* LP
ACAGCCAGCGGTCAAGAC	*hac5* RP
GCAGCCTGCTCACTTGATATC	*taf1* LP
TTCCACTTGGGTTGAACACTC	*taf1* RP
CCCACGAGGTGTATATCATGG	*fve* LP
TTACCTGCAATGTTCCACCTC	*fve* RP
ATTTTGCCGATTTCGGAAC	LBb1.3 (SALK Lines LB)
TAGCATCTGAATTTCATAACCAATCTCGATACAC	LB3 (SAIL Lines LB)
**Primer sequences for RT-PCR/qRT-PCR**	**Primer Name**
GGCTGAAGAGGTTGTTGAGGAAC	*HY5*-Fw
AGCATCTGGTTCTCGTTCTGAAGA	*HY5*-rev
GTGAGCACAAAGTTTAGCGAC	*ELIP.1* Fw
ACTTGGACTCAACGCTTATGC	*ELIP.1* Rev
GTATTGTGCTGGATTCTGGTG	*ACT2* Fw
GAGGTAATCAGTAAGGTCACG	*ACT2* Rev
CAGACAGGACATCGTGGTGGT	*CHS* Fw
ACATGAGTGATCTTTGACTTGG	*CHS* Rev

GTGAGGAGGTAGACGTTGAAAGACT	*CRY3 Fw*
AACTGAGTGTAGACATCAGGCAAGT	*CRY3 Rev*
GAGAGCAAATGGGTGTTGATGTGAA	*WAKL8 Fw*
TTGCCTATCCCAAAAGGGTAAGGAA	*WAKL8 Rev*

Table S2 List of the primers used in this study

Chromosome	TAIR Annotation	Name	Description	Enrichment (+) vs decrease (-) in acetylation following UV-B
Chr1	AT1G01520		Homeodomain-like superfamily protein	+
Chr1	AT1G02340	RSF1|REP1|HFR1|FBI1	basic helix-loop-helix (bHLH) DNA-binding superfamily protein	+
Chr1	AT1G02820		Late embryogenesis abundant 3 (LEA3) family protein	+
Chr1	AT1G06430		FTSH Protease 8	+
Chr1	AT1G06440		Ubiquitin carboxyl-terminal hydrolase family protein	+
Chr1	AT1G09490		NAD(P)-binding Rossmann-fold superfamily protein	+
Chr1	AT1G12370	PHR1|UVR2	photolyase 1	+
Chr1	AT1G13350		Protein kinase superfamily protein	+
Chr1	AT1G16730		unknown protein 6	+
Chr1	AT1G17050		solanesyl diphosphate synthase 2	+
Chr1	AT1G19880		Regulator of chromosome condensation (RCC1) family protein	+
Chr1	AT1G23550		similar to RCD one 2	+
Chr1	AT1G24580		RING/U-box superfamily protein	+
Chr1	AT1G49405		Uncharacterised protein family (UPF0497)	+
Chr1	AT1G53570		mitogen-activated protein kinase kinase kinase 3	+
Chr1	AT1G53580	GLX2-3|ETHE1|GLY3	glyoxalase II 3	+
Chr1	AT1G61610		S-locus lectin protein kinase family protein	+
Chr1	AT1G62710		beta vacuolar processing enzyme	+
Chr1	AT1G64500		Glutaredoxin family protein	+
Chr1	AT1G64780	AMT1;2|ATAMT1;2	ammonium transporter 1;2	+
Chr1	AT1G66730	LIG6|AtLIG6	DNA LIGASE 6	+
Chr1	AT1G68830		STT7 homolog STN7	+
Chr1	AT1G73655		FKBP-like peptidyl-prolyl cis-trans isomerase family protein	+
Chr1	AT1G79270	ECT8	evolutionarily conserved C-terminal region 8	+

Chromosome	TAIR Annotation	Name	Description	Enrichment (+) vs decrease (-) in acetylation following UV-B
Chr1	AT1G80930		MIF4G domain-containing protein / MA3 domain-containing protein	+
Chr2	AT2G15020		unknown protein	+
Chr2	AT2G16365		F-box family protein	+
Chr2	AT2G21960		unknown protein	+
Chr2	AT2G21970		stress enhanced protein 2	+
Chr2	AT2G24540	AFR	Galactose oxidase/kelch repeat superfamily protein	+
Chr2	AT2G25450		2-oxoglutarate (2OG) and Fe(II)-dependent oxygenase superfamily protein	+
Chr2	AT2G29350		senescence-associated gene 13	+
Chr2	AT2G29460	GST22|GSTU4|ATGSTU4	glutathione S-transferase tau 4	+
Chr2	AT2G31370		Basic-leucine zipper (bZIP) transcription factor family protein	+
Chr2	AT2G31380		salt tolerance homologue	+
Chr2	AT2G31750.1	UGT74D1	UDP-glucosyl transferase 74D1	+
Chr2	AT2G33380	RD20|CLO-3	Caleosin-related family protein	+
Chr2	AT2G34080		Cysteine proteinases superfamily protein	+
Chr2	AT2G35920		RNA helicase family protein	+
Chr2	AT2G36750		UDP-glucosyl transferase 73C1	+
Chr2	AT2G37240		Thioredoxin superfamily protein	+
Chr2	AT2G37970		SOUL heme-binding family protein	+
Chr2	AT2G37975	Yos1-like protein		+
Chr2	AT2G40004		unknown protein	+
Chr2	AT2G40010		Ribosomal protein L10 family protein	+
Chr2	AT2G40130		Double Clp-N motif-containing P-loop nucleoside triphosphate hydrolases superfamily protein	+
Chr2	AT2G40460		Major facilitator superfamily protein	+
Chr2	AT2G42540	COR15|COR15A	cold-regulated 15a	+

Chromosome	TAIR Annotation	Name	Description	Enrichment (+) vs decrease (-) in acetylation following UV-B
Chr2	AT2G46830	CCA1	circadian clock associated 1	+
Chr2	AT2G47170	ARF1A1C	Ras-related small GTP-binding family protein	+
Chr2	AT2G47180	galactinol synthase 1		+
Chr2	AT2G47770	AT2G47770.1	TSPO(outer membrane tryptophan-rich sensory protein)-related	+
Chr3	AT3G01060		unknown protein	+
Chr3	AT3G04080	ATAPY1|APY1	APYRASE2	+
Chr3	AT3G09450		CONTAINS InterPro DOMAIN/s: Fusaric acid resistance protein, conserved region (InterPro:IPR006726)	+
Chr3	AT3G09580		FAD/NAD(P)-binding oxidoreductase family protein	+
Chr3	AT3G09590		CAP (Cysteine-rich secretory proteins, Antigen 5, and Pathogenesis-related 1 protein) superfamily protein	+
Chr3	AT3G09600		Homeodomain-like superfamily protein	+
Chr3	AT3G10340	PAL4	phenylalanine ammonia-lyase 4	+
Chr3	AT3G10910		RING/U-box superfamily protein	+
Chr3	AT3G10912		conserved peptide upstream open reading frame 63	+
Chr3	AT3G14680	CYP72A14	cytochrome P450, family 72, subfamily A, polypeptide 14	+
Chr3	AT3G14770		Nodulin MtN3 family protein	+
Chr3	AT3G17609	HYH	HY5-homologue	+
Chr3	AT3G21560	UGT84A2	UDP-Glycosyltransferase superfamily protein	+
Chr3	AT3G21890		B-Box like Zinc Finger protein	+
Chr3	AT3G22840	ELIP1|ELIP	Chlorophyll A-B binding family protein	+
Chr3	AT3G24170	ATGR1|GR1	glutathione-disulfide reductase	+
Chr3	AT3G24180		Beta-glucosidase, GBA2 type family protein	+
Chr3	AT3G24740		Protein of unknown function (DUF1644)	+
Chr3	AT3G27170		CLC-B|ATCLC-B	+

Chromosome	TAIR Annotation	Name	Description	Enrichment (+) vs decrease (-) in acetylation following UV-B
Chr3	AT3G48460		GDSL-like Lipase/Acylhydrolase superfamily protein	+
Chr3	AT3G48700	ATCXE13|CXE13	carboxyesterase 13	+
Chr3	AT3G51240	F3'H|F3H|TT6	flavanone 3-hydroxylase	+
Chr3	AT3G52740		unknown protein	+
Chr3	AT3G54510		Early-responsive to dehydration stress protein (ERD4)	+
Chr3	AT3G56290		unknown	+
Chr3	AT3G56890		F-box associated ubiquitination effector family protein	+
Chr3	AT3G57020		Calcium-dependent phosphotriesterase superfamily protein	+
Chr3	AT3G57030		Calcium-dependent phosphotriesterase superfamily protein	+
Chr3	AT3G57520	SIP2|AtSIP2	seed imbibition 2	+
Chr3	AT3G59390		unknown	+
Chr3	AT3G60110		DNA-binding bromodomain-containing protein	+
Chr3	AT3G61220		NAD(P)-binding Rossmann-fold superfamily protein	+
Ch4	AT4G00050		basic helix-loop-helix (bHLH) DNA-binding superfamily protein	+
Ch4	AT4G05100		myb domain protein 74	+
Ch4	AT4G08870		Arginase/deacetylase superfamily protein	+
Ch4	AT4G12320		cytochrome P450, family 706, subfamily A, polypeptide 6	+
Ch4	AT4G14690	ELIP2	Chlorophyll A-B binding family protein	+
Ch4	AT4G16690	MES16|ATMES16	methyl esterase 16	+
Ch4	AT4G27020			+
Ch4	AT4G27030	FADA|FAD4	fatty acid desaturase A	+
Ch4	AT4G27290		S-locus lectin protein kinase family protein	+
Ch4	AT4G28280	LLG3	LORELEI-LIKE-GPI ANCHORED PROTEIN 3	+
Ch4	AT4G28300		Protein of unknown function (DUF1421)	+
Ch4	AT4G29910	ORC5|ATORC5	origin recognition complex protein 5	+

Chromosome	TAIR Annotation	Name	Description	Enrichment (+) vs decrease (-) in acetylation following UV-B
Ch4	AT4G31860		Protein phosphatase 2C family protein	+
Ch4	AT4G31870	GPX7|ATGPX7	glutathione peroxidase 7	+
Ch4	AT4G34140		D111/G-patch domain-containing protein	+
Ch4	AT4G35090	CAT2	CATALASE	+
Ch4	AT4G35250		NAD(P)-binding Rossmann-fold superfamily protein	+
Ch4	AT4G36050		endonuclease/exonuclease/phosphatase family protein	+
Ch4	AT4G37150	MES9|ATMES9	methyl esterase 9	+
Ch4	AT4G37760	SQE3	squalene epoxidase 3	+
Ch5	AT5G05140		Transcription elongation factor (TFIIS) family protein	+
Ch5	AT5G11260	HY5	Basic-leucine zipper (bZIP) transcription factor family protein	+
Ch5	AT5G13930	CHS	Chalcone and stilbene synthase family protein	+
Ch5	AT5G17300	RVE1	Homeodomain-like superfamily protein	+
Ch5	AT5G17310		UDP-glucose pyrophosphorylase 2	+
Ch5	AT5G17780		alpha/beta-Hydrolases superfamily protein	+
Ch5	AT5G19850		alpha/beta-Hydrolases superfamily protein	+
Ch5	AT5G19855		Chaperonin-like RbcX protein	+
Ch5	AT5G19870		Family of unknown function (DUF716)	+
Ch5	AT5G23730		Transducin/WD40 repeat-like superfamily protein	+
Ch5	AT5G24120		sigma factor E	+
Ch5	AT5G24150		FAD/NAD(P)-binding oxidoreductase family protein	+
Ch5	AT5G24155		FAD/NAD(P)-binding oxidoreductase family protein	+
Ch5	AT5G24880		BEST Arabidopsis thaliana protein match is: calmodulin-binding protein-related	+
Ch5	AT5G28840		GDP-D-mannose 3',5'-epimerase	+
Ch5	AT5G37550		unknown protein	+
Ch5	AT5G39080		HXXXD-type acyl-transferase family protein	+

Chromosome	TAIR Annotation	Name	Description	Enrichment (+) vs decrease (-) in acetylation following UV-B
Ch5	AT5G42760		Leucine carboxyl methyltransferase	+
Ch5	AT5G43860		chlorophyllase 2	+
Ch5	AT5G50360		unknown protein	+
Ch5	AT5G53970		Tyrosine transaminase family protein	+
Ch5	AT5G55570		unknown protein	+
Ch5	AT5G57670		Protein kinase superfamily protein	+
Ch5	AT5G58340		myb-like HTH transcriptional regulator family protein	+
Ch5	AT5G58760	DDB2	damaged DNA binding 2	+
Ch5	AT5G58770		Undecaprenyl pyrophosphate synthetase family protein	+
Ch5	AT5G61070	ATHDA18|HDA18	histone deacetylase of the RPD3/HDA1 superfamily 18	+
Ch5	AT5G61640	PMSR1|ATMSRA1	peptidemethionine sulfoxide reductase 1	+
Ch5	AT5G65280		GCR2-like 1	+
Chr1	AT1G19340		Methyltransferase MT-A70 family protein	-
Chr1	AT1G80280		alpha/beta-Hydrolases superfamily protein	-
Chr2	AT2G42380	BZIP34|ATBZIP34	Basic-leucine zipper (bZIP) transcription factor family protein	-
Chr2	AT2G43010	PIF4	phytochrome interacting factor 4	-
Chr2	AT2G44735		BEST Arabidopsis thaliana protein match is: F-box family protein	-
Chr2	AT2G44740	CYCP4;1	cyclin p4;1	-
Ch5	AT5G25190		Integrase-type DNA-binding superfamily protein	-

Table S3 Complete List of the genetic loci identified by ChIPDiff, that display differential H3K9,14diac enrichment levels following UV-B illumination. The last 7 rows (orange) show the loci that were found to display UVR8-dependent, UV-B induced decrease in acetylation
